# Reduced task adaptation and contextual awareness in autistic adults during facial emotion recognition: evidence from mixed-effects modeling and automated facial analysis

**DOI:** 10.1186/s13229-026-00711-6

**Published:** 2026-03-11

**Authors:** Simon Kirsch, Hanna Drimalla, William Saakyan, Bastian Elmar Alexander Sajonz, Justus Gritzmann, Simon Maier, Thomas Fangmeier, Muyu Lin, Simón Guendelman, Christian Kaufmann, Isabel Dziobek, Ludger Tebartz van Elst

**Affiliations:** 1 Department of Psychiatry and Psychotherapy, Faculty of Medicine, Medical Center-University of Freiburg, Freiburg, Germany; 2 Humboldt-Universität zu Berlin, Institute of Psychology, Berlin, Germany; 3 Center for Cognitive Interaction Technology (CITEC), Bielefeld University, Bielefeld, Germany; 4 Department of Stereotactic and Functional Neurosurgery, Faculty of Medicine, Medical Center – University of Freiburg, Freiburg, Germany; 5 Berlin School of Mind and Brain, Humboldt-Universität zu Berlin, Berlin, Germany

**Keywords:** Autism, Facial expression, Emotion recognition, Adults, Automated analysis, Mixed effects modelling, Computer vision

## Abstract

**Background:**

Despite significant advances in understanding facial emotion recognition (FER) in autistic adults in recent decades, the mechanisms underlying FER difficulties in individuals with autism remain unclear, with inconsistent findings across studies. A key limitation may be the reliance on aggregate accuracy scores, which overlook item- and subject-level variability. Here, we investigated the effects of task adaptation and stimulus properties on FER performance in autistic and non-autistic adults using mixed-effects modelling.

**Methods:**

A total of 120 autistic and 116 non-autistic participants completed the Berlin Emotion Recognition Test 2. Performance was analyzed on a trial-by-trial basis, considering trial number, stimulus properties—derived from automated facial analysis—and their interactions with diagnostic group. Response times were analyzed using mixed-effects linear regression models, while accuracy was analyzed using mixed-effects logistic regression models.

**Results:**

Compared with non-autistic participants, autistic participants demonstrated lower overall accuracy and slower responses, accompanied by significantly reduced task adaptation. Contextual ambiguity of stimulus faces moderated group differences in FER accuracy, with non-autistic subjects showing greater use of contextual information. Social-cognitive traits further moderated the effect of contextual ambiguity in autistic subjects.

**Limitations:**

Our findings are specific to the design and stimulus material of the Berlin Emotion Recognition Test 2 and may not generalize to other FER tasks. Furthermore, our sample did not include individuals with intellectual disabilities, limiting generalizability across the autism spectrum. Lastly, the reliability of stimulus property estimates derived from automated facial analysis may require validation on a larger sample of stimulus faces.

**Conclusions:**

Our findings reveal that differences in task adaptation and contextual cue processing underlie FER performance differences in individuals with autism, emphasizing the importance of participant- and item-level analysis. These results may inform future study designs and highlight the advantages of integrating automated FER with mixed-effects modeling in autism research.

**Supplementary information:**

The online version contains supplementary material available at 10.1186/s13229-026-00711-6.

## Background

Difficulties in social communication and interaction are a defining characteristic of autism spectrum disorder (ASD) [1]. Emotion recognition is central to social interaction [2] and relies on the integration of multiple social signals across modalities [3]. Facial emotion recognition has long been linked to ASD [4] and may contribute to broader social communication challenges in autistic individuals [5]. However, findings on FER in individuals with ASD remain inconsistent, with some studies finding intact FER in individuals with ASD and others finding profound deficits [6].

A recent meta-analysis concluded that ASD is associated with a general FER deficit across all basic emotions and that this deficit is more pronounced in individuals with ASD than in other clinical populations [7]. The authors also reported that emotion complexity, holistic processing and task characteristics moderate the effects of ASD on emotion recognition performance. Despite extensive research, most studies have used aggregated accuracy scores, potentially overlooking variability at both the participant and item levels. Inconsistent FER findings in ASD may therefore reflect individual heterogeneity and differences in task demands [8].

Generalized linear mixed-effects models (GLMMs) with a logit link function allow researchers to analyze binary outcomes, such as correct vs. incorrect FER responses, while controlling for repeated measures [9]. GLMMs thereby allow for a more fine-grained examination of how FER performance unfolds over time and across heterogeneous stimulus material. Task demands in FER experiments may vary both over time – reflecting processes such as adaptation or exhaustion – and across stimuli, which differ in the amount of clarity of emotional information they convey. Accordingly, we examined (a) whether FER performance changes during the task and whether time trends differ between ASD and non-autistic comparison (NC) subjects and (b) whether stimulus salience affects FER performance differently between ASD and NC individuals. Together, these analyses allow us to assess systematic performance differences that are obscured when FER is assessed using aggregated accuracy scores, thereby offering a framework for understanding heterogeneity and inconsistent findings in prior research.

### Processing styles

Facial expressions are processed both implicitly (automatic, inflexible) and explicitly (conscious, flexible, resource-demanding) [10, 11]. FER tasks typically assess explicit processing, as they explicitly draw attention to the emotional state of the perceived face [11, 12]; however, the automaticity of labeling may vary by proficiency and task difficulty. While for some people, the correct emotion label for a given face may be instantly apparent, others may hesitate and need to assess specific facial features. Even within one individual, certain facial expressions may be obvious, whereas others require conscious processing. Accordingly, processing style may vary both within and between participants in FER experiments. This is particularly important in the context of autism research, where compensatory cognitive strategies, such as featural processing, are often assumed to explain differences in emotion recognition abilities among individuals with ASD [6]. These processing styles may vary dynamically depending on the stimulus and time‑on‑task. Consequently, changes in performance over trials and sensitivity to stimulus characteristics may reflect shifts in the relative contribution of implicit and explicit processing routes.

### Adaptation and exhaustion

As compensatory strategies are considered to be more cognitively demanding [13], autistic individuals may experience a decline in FER performance over the course of the experiment due to exhaustion, particularly when relying on explicit, resource‑demanding processing styles. On the other hand, it may take time for explicit cognitive strategies to become effective in compensating for existing difficulties in FER due to adaptation. These effects could amplify or obscure group differences.

To our knowledge, this is the first study to examine the influence of adaptation or exhaustion effects on FER differences in individuals with ASD by including trial number as a linear predictor variable in GLMMs. By including trial number as a continuous predictor and its interaction with the diagnostic group, we aim to identify whether performance follows a linear trend of improvement or decline and whether this trend differs between autistic and non-autistic participants.

### Influence of stimulus salience

At the item level, different manifestations of emotional expression in faces can affect recognition difficulty due to various factors, such as expression intensity, ambiguity or prototypicality [14]. In this study, we refer to these differences in stimulus faces that affect the recognition of the expressed emotions as “stimulus salience”. Stimulus salience is defined as the amount of information about the intended emotional expression and may include variations in intensity, ambiguity and prototypicality.

The influence of expression intensity on FER differences in ASD has been investigated via morphs of neutral and emotional faces [15] and videos of actors transitioning from a neutral facial expression to an emotional facial expression [16]. Both approaches indicate reduced sensitivity to emotional information in individuals with ASD. However, these approaches presuppose that the maximally expressive emotional face contains the greatest amount of emotional information, a notion that can be undermined by an actor’s unclear portrayal of an emotion. Moreover, both approaches assume an approximately linear relationship between information content and visual changes in the stimulus faces. Although recognition accuracy generally improves with increasing emotional intensity [17], certain prototypical expressions of emotion may be detectable even at relatively low intensities, whereas ambiguous emotional expressions may remain challenging even at higher intensities [14, 18].

Validation studies can control for the quality of emotional expressions in facial stimuli by investigating the FER performance of large samples of non-clinical participants, as done for standardized facial databases [19, 20]. High accuracy scores indicate high levels of stimulus salience. However, despite the effort involved, these studies cannot be used to determine stimulus salience, since low accuracy may depend on various factors, such as intensity, ambiguity and prototypicality. Facial action coding [21] offers a way to quantify the underlying expression parameters of these factors. However, manual annotation of facial expressions is time-consuming and subject to limited inter-rater reliability [22]. Automated FER models may therefore provide an efficient and more objective way to estimate quantitative parameters of stimulus salience.

We used the automated FER model in FaceReader (Version 9, Noldus, Amsterdam, Netherlands) to estimate the probability of each of the six basic emotions being displayed by each stimulus face. These probability values were then used to calculate three metrics of stimulus salience, as detailed in the methods section. FaceReader provides excellent FER performance, outperforming human raters on standardized images under controlled lighting and face angle conditions [23].

### The present study

This study employs mixed-effects modeling to analyze the influence of adaptation/exhaustion and stimulus salience on FER in individuals with ASD. Specifically, it investigates the effects of trial number and stimulus salience—along with their interactions with autism diagnosis—on both accuracy and log-transformed response times (logRT) in the Berlin Emotion Recognition Test 2 (BERT2).

Based on assumptions about compensatory strategies, we hypothesized that autistic subjects would show changes – improvements or declines – in FER performance over the course of the experiment, whereas performance in non-autistic participants was expected to remain relatively stable. With respect to stimulus characteristics, we hypothesized that higher stimulus salience would be associated with better FER performance in both groups. In addition, drawing on evidence for reduced sensitivity to emotional information in ASD [15, 16], we hypothesized that stimulus salience would differentially affect autistic and non-autistic participants, with the strongest performance decrements in autistic individuals expected for stimuli with low salience.

## Methods

### Participants

**Fig. 1:**
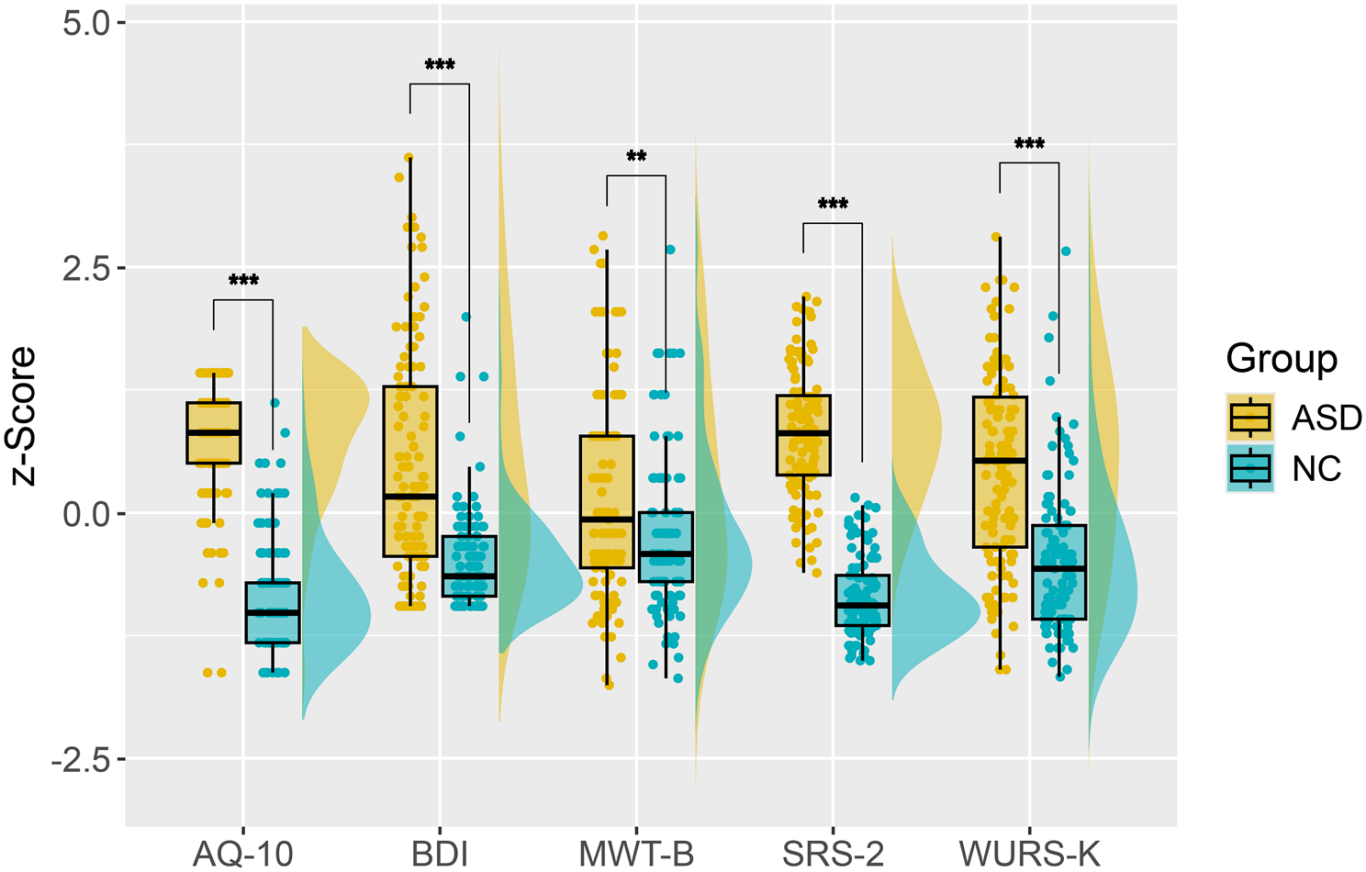
Raincloud plots illustrating the distribution of questionnaire results across the ASD and NC groups. All results were centered and scaled via z-transformation to allow for comparisons of group differences between questionnaires. AQ10, Brief autism Quotient; BDI, Beck Depression Inventory; MWT-B, Mehrfachwahl-Wortschatz-Intelligenztest; SRS-2, Social Responsiveness Scale (self-report), Second Edition; WURS-K, Wender Utah Rating Scale-Kurzform. ** *p* < 0.01. *** *p* < 0.001

We included 120 adult participants with ASD and 116 NCs, with no self-reported history of psychiatric or neurological disorders. ASD participants were recruited from a psychotherapy trial (DRKS-ID: DRKS00017817; see study protocol for details [24]). All had a confirmed autism or Asperger’s syndrome diagnosis (ICD-10 F84.0, F84.1, or F84.5) via the Autism Diagnostic Observation Schedule (ADOS-2) [25] or an experienced psychiatrist using the ICD-10 criteria. The participants had no intellectual disability (IQ ≥ 80) and no acute severe depression. As expected, participants with ASD reported significantly greater levels of depressive symptoms, as measured by the Beck Depression Inventory (BDI), and elevated symptoms of attention-deficit/hyperactivity disorder (ADHD), as assessed via the short version of the Wender Utah Rating Scale (WURS-K), compared to NCs. In addition, the ASD group presented significantly greater verbal intelligence, as assessed with the multiple-choice vocabulary test, version B (MWT-B; see Table 1 and Fig. 1).

**Table 1:** Demographic and psychometric data comparing ASD and NC group participants. Values are presented as n or mean ± SD. Between-group differences in age and psychometric scores were tested via Kruskal‒Wallis H tests due to the non-normality of the distributions. Between-group differences in gender distribution were tested using a χ2 test

Measurement	ASD (n = 120)	NC (n = 111)	Test statistic (df)	n missing
Gender (Female Diverse, Male)	F = 56; D = 3; *M* = 61	F = 49; D = 0; *M* = 62	χ2(2) = 3.13	-
Age (Years)	35.3 (±11.2)	36.7 (±11.4)	H(1) = 0.8	-
AQ10	7.9 (±2.0)	2.6 (±1.9)	H(1)=141.6***	-
SRS-2	105.0 (±25.4)	39.4 (±15.9)	H(1)=159.6***	ASD = 4; NC = 0
WURS-K	36.9 (±13.4)	24.3 (±10.5)	H(1)=51.4***	ASD = 2; NC = 0
BDI	14.0 (±11.1)	4.6 (±4.9)	H(1)=53.4***	ASD = 4; NC = 0
MWT-B	110.1 (±15.7)	103.8 (±11.8)	H(1)=8.45**	ASD = 9;NC = 2

**Significant at *p* < 0.01

***Significant at *p* < 0.001

AQ10, Brief Autism Quotient; ASD, autism spectrum disorder; BDI, Beck Depression Inventory; MWT-B, Mehrfachwahl-Wortschatz-Intelligenztest; NC, nonautistic comparison participant; SRS-2, Social Responsiveness Scale (self-report), Second Edition; WURS-K, Wender Utah Rating Scale-Kurzform

This study was part of a larger project investigating neurobiological and behavioral differences in socio-emotional processing between autistic and non-autistic adults. For a detailed description of the project, please refer to the pre-registration (https://osf.io/p72fz). The study protocol was reviewed and approved by the local ethics committee of the Medical Center – University of Freiburg, Germany.

Five NCs were excluded from the analysis as they exceeded our cut-off of 80 points on the SRS-2, indicating social impairments. This resulted in a final sample size of 231 participants (120 ASD; 111 NC). The sample size for the project was determined by power considerations on the basis of the neurobiological analyses (see pre-registration: https://osf.io/p72fz).

### Procedure

Testing took place during an ~3.5-hour lab session, including MRI and behavioral tasks. In addition, participants completed a battery of online-questionnaires at home. Behavioral tests were administered after the MRI measurements (see Supplementary Material Section 1 for a summary of the measurements and test sequence).

A total of 111 participants (57 ASD; 54 NC) were tested at the Humboldt Universität zu Berlin, and 120 participants (63 ASD; 57 NC) were tested at the University Hospital of Freiburg. For BERT2, participants sat in front of a laptop and received written instructions. After a test run consisting of four trials, the participants were given the opportunity to ask questions. The task took approximately 5–10 minutes.

### Berlin Emotion Recognition Test 2

**Fig. 2:**
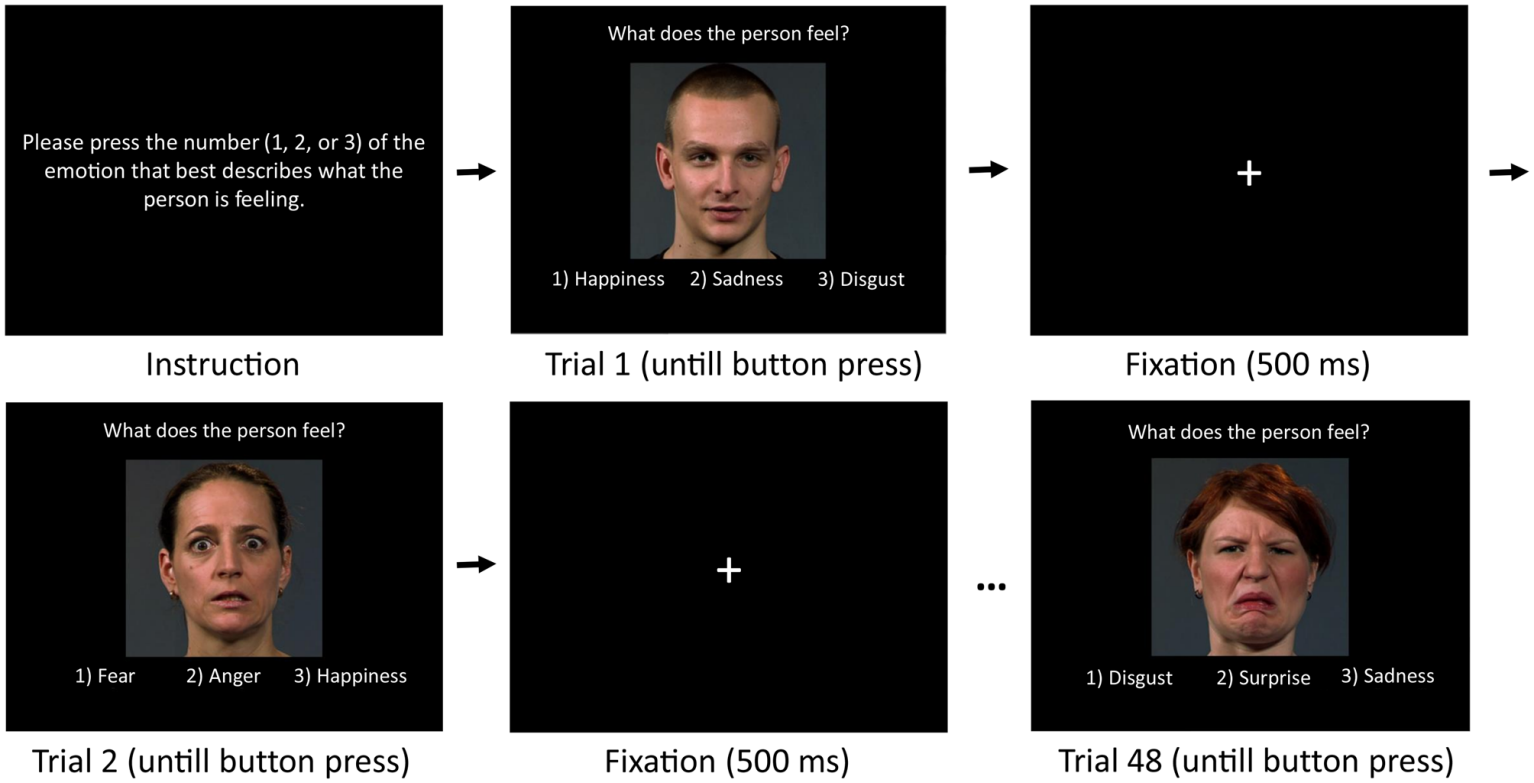
Illustration of the Berlin Emotion Recognition Test 2. Instructions and labels were presented in German and translated into English for illustration purposes

The BERT2 is an updated version of the BERT [26], the only difference being that instead of two choices, it presents three possible emotion labels. It is a computer-based test for FER abilities and involves 48 photographs of professional actors expressing one of the six basic emotions. There are eight pictures per emotion, balanced by gender (male/female). For each picture, three emotion labels are shown (one target, two distractors), and participants were asked to select what the person feels as quickly and accurately as possible, without imposing a fixed time limit (see Fig. 2). Only one choice, the target-label, matches the displayed emotion. The position of the target-label and stimulus order are fully randomized. Stimuli were extracted from videos of actors expressing the target emotions via emotional scripts (e.g., “imagine you received an unexpected present”), starting with a neutral expression. The procedure was followed to obtain naturalistic footage.

In a preliminary study, stimulus pictures were selected from the video material that discriminated best between high- and low-scoring participants to maximize the sensitivity to difficulties in FER. A detailed description of the task development and the task itself can be found online [27].

Within our data, we found that non-autistic participants did not exceed chance level for 4 stimuli, when comparing the hit-rate with the closest distractor’s false positive-rate. We therefore excluded these stimuli from our analyses, resulting in a final set of 44 facial expressions.

### Social Responsiveness Scale (SRS-2)

**Fig. 3:**
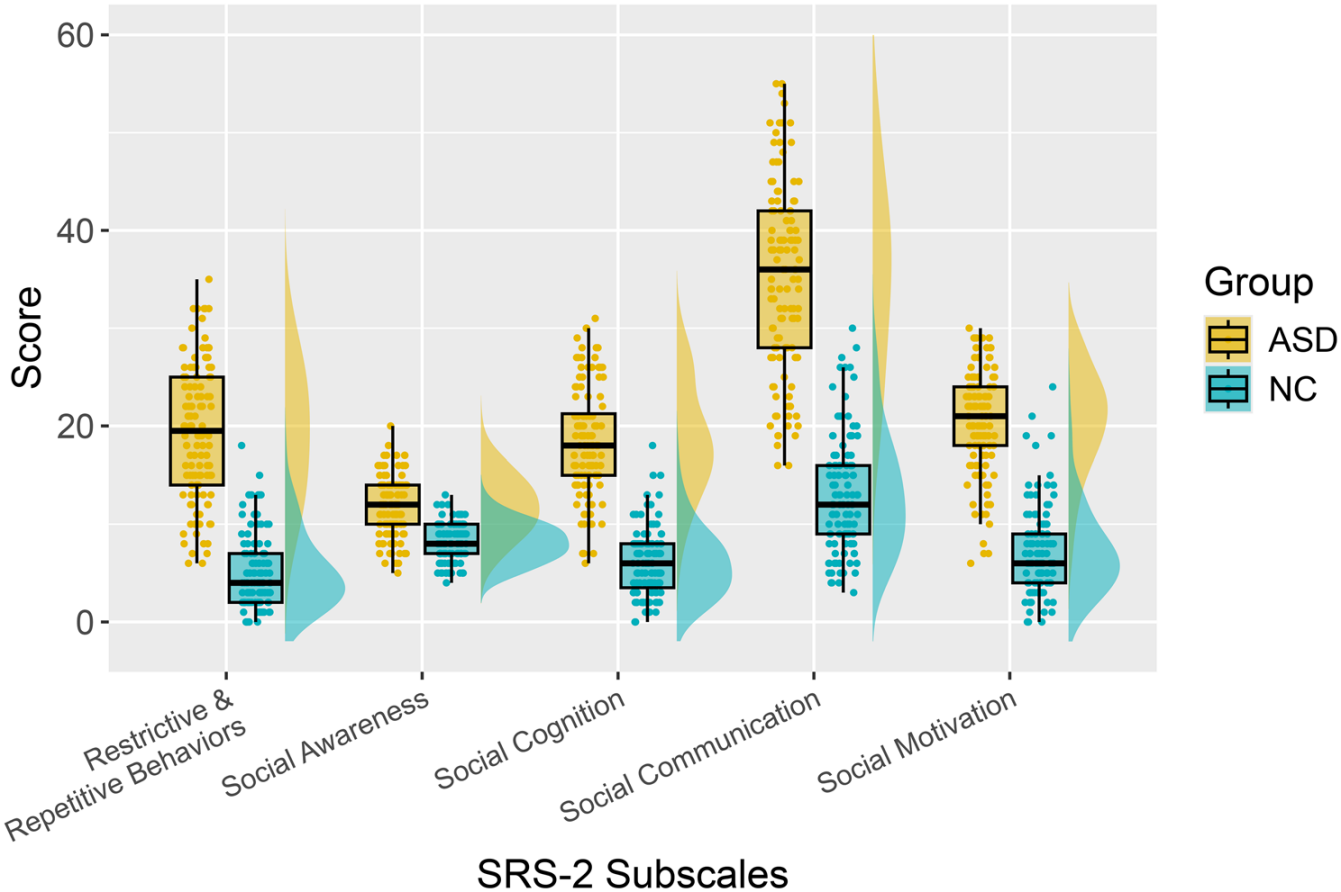
Raincloud plots illustrating the distribution of SRS subscale scores across the ASD and NC groups

The SRS-2 is a 65-item questionnaire measuring symptoms of ASD. In this study, we used the German adaptation of the adult self-report version of the SRS-2. All items are rated on a four-point frequency scale (1 = never true; 4 = almost always true). The sum of all item scores comprises the total score. Each item belongs to one of five subscales, including social awareness, social cognition, social communication, social motivation and restrictive and repetitive behaviors. Figure 3 shows the distribution of SRS-2 subscale scores across groups.

### Automated facial emotion recognition

Each of the 48 BERT2 stimulus faces was analyzed via the DeepFace facial expression classification method [28] implemented in FaceReader 9. For each stimulus face, FaceReader provides probability values for all basic emotions. From these, we computed three different measures of stimulus salience:

1. **Target-probability** reflects the model’s estimated probability of the intended target emotion (from 0 to 1). On a psychological level, the target-probability may indicate the clarity of the intended emotional expression, which can be determined by the intensity or prototypicality of that expression, irrespective of any other possible emotions.
2. **General ambiguity** was calculated using Shannon entropy. Entropy describes the uncertainty in the model’s probability space over all basic emotions: if the model assigns similar probabilities to multiple emotions, entropy is high, indicating uncertainty in classification. Conversely, if one emotion has a dominant probability while the others are low, entropy is low, reflecting an unambiguous emotional expression. The formula for entropy is $H = - \mathop \sum \nolimits {p_i} lo{g_2}({p_i})$, where ${p_i}$ represents the probability assigned to each of the six emotions.
3. **Contextual ambiguity** was quantified using the target-distractor difference, defined as the difference between the probability assigned to the target emotion and the higher of the two probabilities assigned to the distractor emotions. Larger target-distractor differences indicate lower contextual ambiguity and, consequently, greater confidence in the correct response, whereas smaller or negative values reflect higher contextual ambiguity, indicating uncertainty or potential misclassification. The target-distractor difference was calculated as: $Target -$ $Distractor$ $Difference$ $= {p_{target}}$ $- {p_{distractor}}$ where ${p_{target}}$ is the target-probability and ${p_{distractor}}$ is the probability of the most likely distractor.

### Statistical analysis

Accuracy and RTs were recorded; RTs were limited to correct responses and log-transformed to account for skewed distribution. All data were analyzed using R (version 4.2.2). We analyzed the effects of group, trial number, target-probability, general ambiguity, contextual ambiguity and SRS-2 scores using LMMs for RTs [29] and logit GLMMs for accuracy [9]. Participant and item variability was controlled for via random intercepts. Categorical variables were deviation coded, meaning that factor levels were compared to the grand mean. All numerical variables were scaled and centered. Where appropriate, post-hoc models were conducted to interpret group-specific effects, using Holm correction for multiple comparisons. The full code, along with tests for modeling assumptions, can be found in the Supplementary Material (Section 4).

## Results

### FaceReader results

Emotional expressions were classified by extracting the estimated probability of each basic emotion category from the automated FER model. For each face, the emotion corresponding to the highest probability was selected as the predicted emotional expression. According to this procedure, FaceReader correctly classified 37 out of 44 emotional faces, resulting in a classification accuracy of 84.09%. When only the probabilities of the three response options were taken into account (target probability and both distractor probabilities), the accuracy increased to 86.36% (adjusted classification accuracy). The emotion probabilities and classifications for all stimulus faces can be found in Supplementary Table S2.1.

### Main effect of group

#### Accuracy

**Fig. 4:**
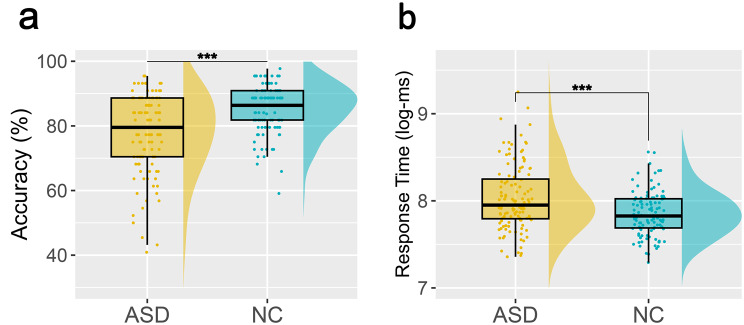
Raincloud plots illustrating task performance differences between ASD and NC. **a**) group differences in terms of accuracy. **b**) group differences in terms of log-transformed response times (logarithmized milliseconds). *** *p* < 0.001

First, we employed a GLMM to investigate the main effect of group on FER accuracy (see Supplementary Material Section 4 for all model specifications). The model revealed a significant negative effect of autism diagnosis (log-odds = −0.27; ${\chi ^2}\left( 1 \right)$ = 33.9; *p* < 0.001), indicating lower overall accuracy in individuals with ASD (see Fig. 4a). In terms of probabilities, the model estimated mean accuracies of 87.82% and 80.85% for NC and ASD, respectively, giving a mean difference of 6.97%.

#### Response time

The respective LMM predicting logRT showed a significant positive effect of autism diagnosis

(β = 0.08; F (1,228)=15.80; *p* < 0.001), indicating higher RTs in ASD (see Fig. 4b). In terms of milliseconds, the model estimated a mean RT of 2505 ms for the NC group and a mean RT of 2963 ms for the ASD group, giving a mean difference of 457 ms.

### Effects of trial number

#### Accuracy

**Fig. 5:**
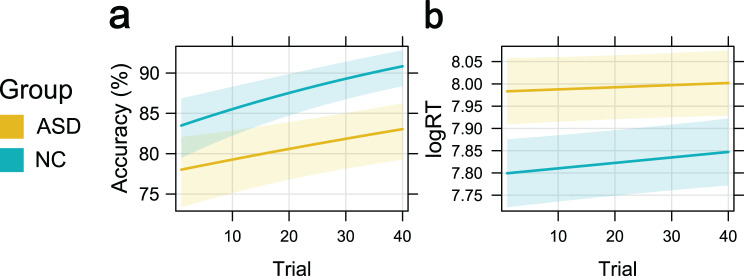
Effects plots illustrating the predictor effect of trial number on **a**) FER accuracy and **b**) log-transformed response times for ASD and NC. Predictor effects, including between-group interactions, were extracted from the full models. The shaded area around the effect line represents the 95% confidence interval of the estimated effect

A GLMM, including trial number and group as interacting variables, revealed significant main effects of trial number (log-odds = 0.16; ${\chi ^2}\left( 1 \right)$ = 34.80; *p* < 0.001) and group (log-odds = −0.27; ${\chi ^2}\left( 1 \right)$ = 35.0; *p* < 0.001) as well as a significant interaction of both variables (log-odds = 0.06; ${\chi ^2}\left( 1 \right)$ = 4.34; *p* = 0.037; see Fig. 5a).

Post-hoc models confirmed significant effects of trial number within both groups. For NC, the model estimates indicate an average first-trial accuracy of 84.08% and an average last-trial accuracy of 91.66% (log-odds = 0.22; ${\chi ^2}\left( 1 \right)$ = 25.48; ${p_{corrected}}$ < 0.001). For ASD, the average estimated accuracy increased from 77.77% to 83.23% across trials (log-odds = 0.10; ${\chi ^2}\left( 1 \right)$ = 8.57; ${p_{corrected}}$ = 0.003). Thus, while both groups showed significant adaptation across the task, the ASD group exhibited lower overall accuracy and reduced adaptation over time.

#### Response time

The respective LMM predicting logRT revealed significant main effects of trial number (β = 0.01; F (1,7997)=6.51; *p* < 0.011) and group (β = 0.08; F(1,228) = 15.86; *p* < 0.001) but no significant interaction between the two variables (β < 0.01; F(1,7997) = 1.25; *p* = 0.264; see Figure 5b). These results indicate that response times generally increased over trials, with ASD participants responding more slowly on average than NC participants did. However, there was no statistical evidence that the rate of change in response times differed between groups.

### Effects of stimulus salience

#### Accuracy

**Fig. 6:**
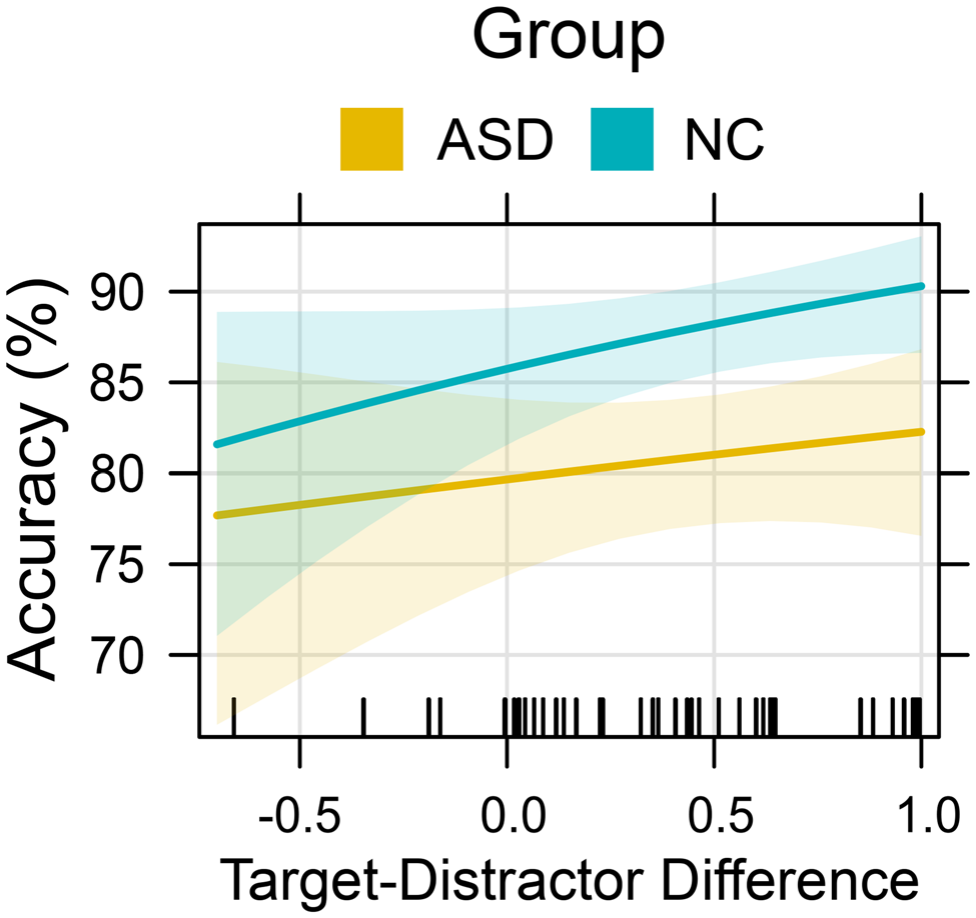
Effects plot illustrating the predictor effect of the target-distractor difference, on the FER accuracy for ASDs and NCs. Larger target-distractor differences indicate lower contextual ambiguity and vice versa. The predictor effect, including between-group interactions, was extracted from the full model. The shaded area around the effect line represents the 95% confidence interval of the estimated effect. The vertical rug marks on the x-axis indicate the observed values of the predictor variable

The effects of stimulus salience were investigated using three separate GLMMs, each including one of the three stimulus salience variables (target probability, general ambiguity and contextual ambiguity) and group as an interacting factor. None of the stimulus salience variables revealed a significant main effect on FER accuracy (see Supplementary Material Sections 4.3–4.5). The only variable that revealed a significant interaction with the group variable was contextual ambiguity (log-odds = −0.13; ${\chi ^2}\left( 1 \right)$ = 4.36; *p* = 0.037). The directionality of the interaction indicated that autistic subjects benefitted less from reductions in contextual ambiguity (i.e., greater target-distractor difference; see Fig. 6), although a group-specific post-hoc model did not show a significant main effect of contextual ambiguity for NC (log-odds = 0.45; ${\chi ^2}\left( 1 \right)$ = 2.38; *p* = 0.123).

#### Response times

**Fig. 7:**
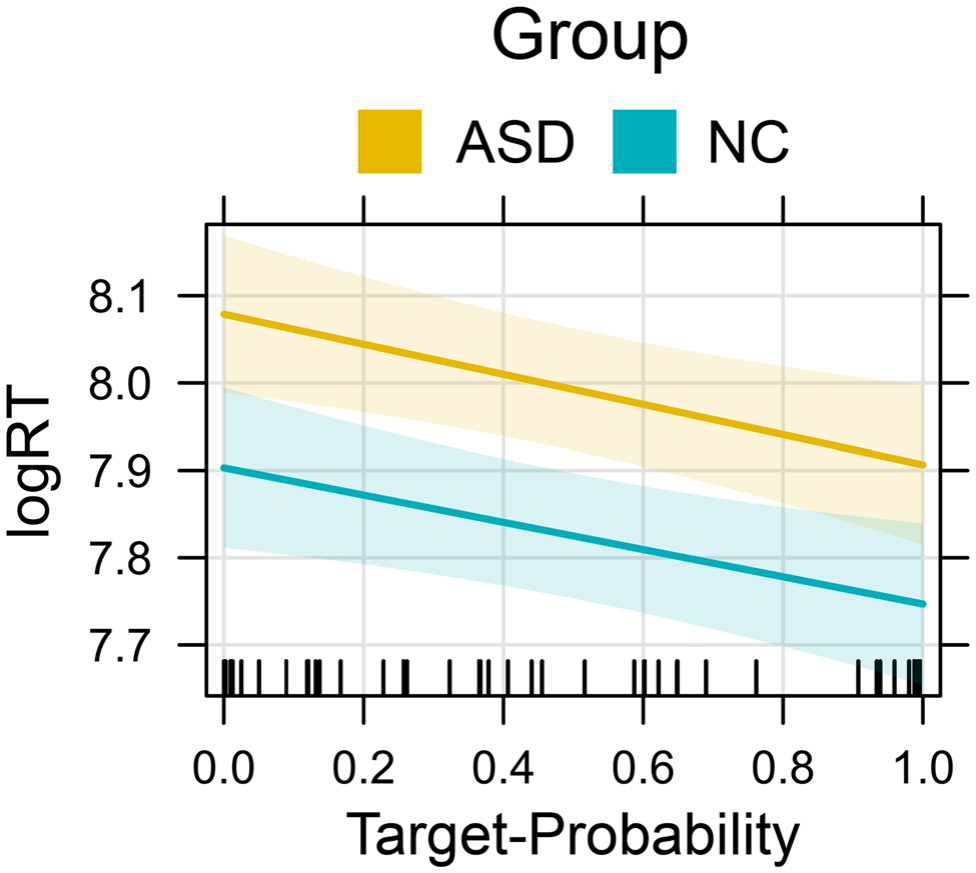
Effects plot illustrating the predictor effect of target-probability on log-transformed response times for ASD and NC. The shaded area around the effect line represents the 95% confidence interval of the estimated effect. Vertical rug marks on the x-axis indicate observed values of the predictor variable

The respective LMMs predicting logRT revealed significant main effects of target probability (β = −0.16; F (1,42)=8.08; *p* = 0.007; see Figure 7) and contextual ambiguity (β = −0.13; F(1,42) = 6.77; *p* = 0.013). In contrast to accuracy, no significant interactions were found between stimulus salience and the group variable at the level of response time. These results indicate that both the ASD and NC group responded more slowly to stimuli with lower target probabilities and higher contextual ambiguity (i.e., low delta). General ambiguity did not significantly affect RTs in either group.

### Effects of social responsiveness

#### Accuracy

We tested the effects of the Social Responsiveness Scale, Second Edition self-report questionnaire [30, 31], and all subscale scores on FER performance in individual GLMMs. As the SRS-2 is designed to measure deficits in social behavior associated with ASD [32] and the two groups therefore present significantly different scores (see Fig. 3), the effects were analyzed via group-specific models only.

**Fig. 8:**
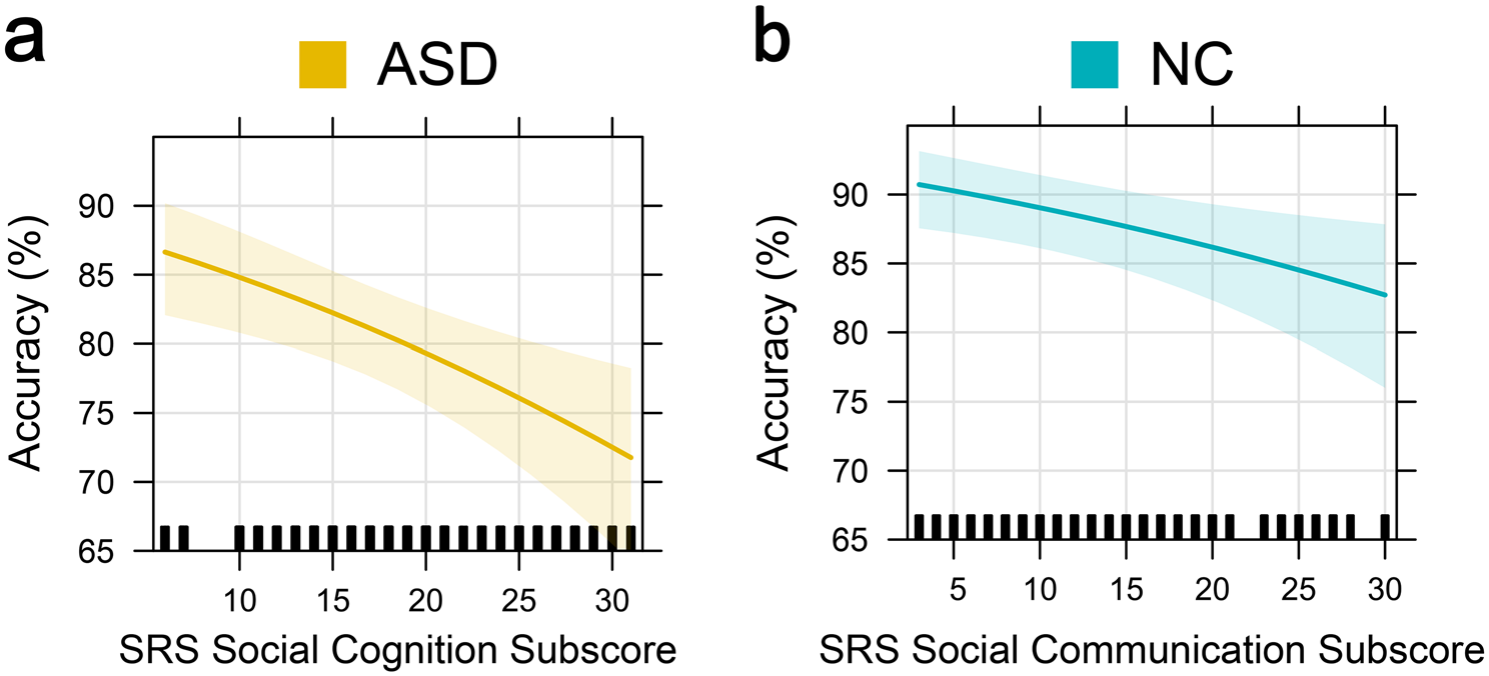
Effects plots illustrating the predictor effects of the SRS subscales, most significantly affecting FER accuracy in ASD and NC. Predictor effects were extracted from separate models for each group. **a**) effect of the SRS social cognition subscore on FER accuracy in ASD. **b**) effect of the SRS social communication subscore on FER accuracy in NC. The shaded area around the effect line represents the 95% confidence interval of the estimated effect. The vertical rug marks on the x-axis indicate the observed values of the predictor variable

Social responsiveness, as measured by the SRS-2, was a significant predictor of FER difficulties in ASD (log-odds = −0.18; ${\chi ^2}\left( 1 \right)$ = 7.63; *p* = 0.006), and in NC patients (log-odds = −0.12; ${\chi ^2}\left( 1 \right)$ = −2.06; *p* = 0.039). Within ASD, social awareness, social cognition and social communication showed a significant effect on emotion recognition accuracy, with social cognition revealing the largest estimated effect on FER accuracy (log-odds = −0.21;${\chi ^2}\left( 1 \right)$ = 10.90; *p* < 0.001; see Fig. 8a). Within NC, only social communication showed a significant effect on emotion recognition accuracy (log-odds = −0.16; ${\chi ^2}\left( 1 \right)$ = 7.34; *p* = 0.007; see Fig. 8b).

#### Response time

In line with the accuracy analyses, SRS effects on RT were analyzed using group-specific LMMs only, due to the significantly different distributions between groups. In none of the groups did SRS scores or any of the subscale scores significantly predict RT.

### Interaction effects on social-cognitive traits in individuals with ASD

We hypothesized that emotion recognition in individuals with ASD would be strongly influenced by social-cognitive difficulties. This theoretical assumption was supported by our results from the analyses of the SRS-2 subscales. We therefore examined the interaction of social-cognitive traits (as measured by the SRS-2 Social Cognition subscale) with the effects of contextual ambiguity and trial number within ASD.

**Fig. 9:**
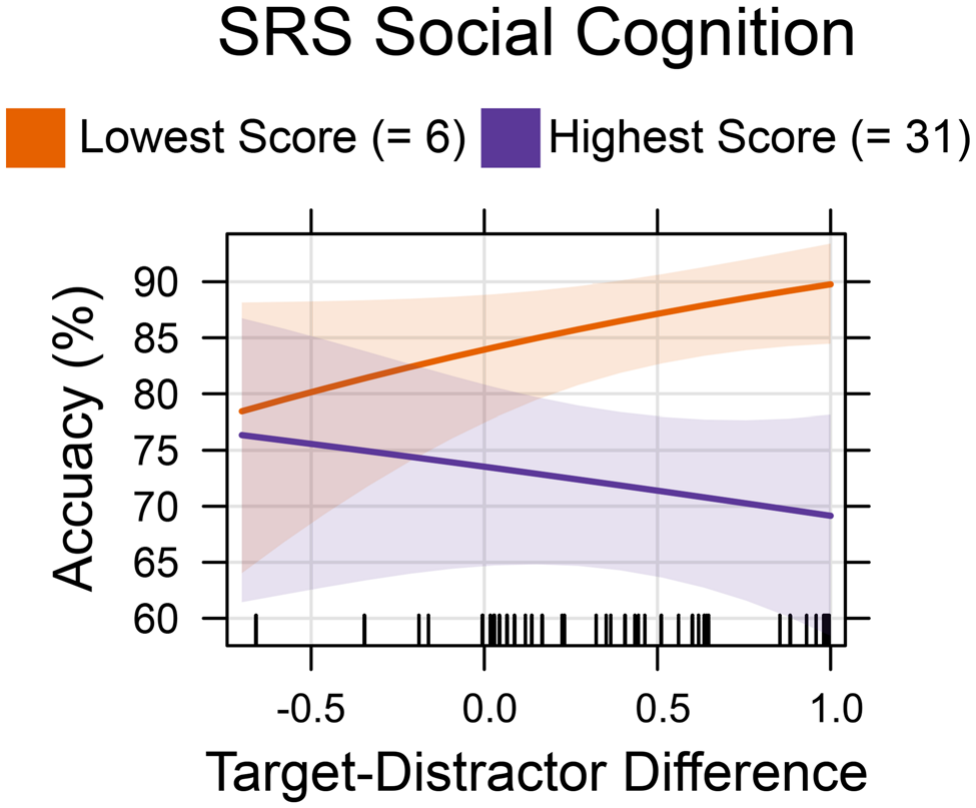
Effects plot illustrating the interaction effect of social-cognitive ability (SRS social cognition subscore) and target-distractor difference on FER accuracy in ASD. Low SRS social cognition subscores indicate good social-cognitive abilities. Low target-distractor differences indicate high contextual ambiguity. For illustration purposes, we present the predicted effect of cognitive ambiguity on FER accuracy at the lowest and highest recorded SRS social cognition subscores. The shaded area around the effect line represents the 95% confidence interval of the estimated effect. The vertical rug marks on the x-axis indicate the observed values of the predictor variable

The first GLMM showed a significant main effect of social-cognitive traits (log-odds = −0.14; ${\chi ^2}\left( 1 \right)$ = 3.88; *p* = 0.049), as well as a significant interaction of social-cognitive traits and contextual ambiguity (log-odds = −0.17; ${\chi ^2}\left( 1 \right)$ = 4.16; *p* = 0.041), indicating that autistic subjects with higher social-cognitive traits benefited less from lower contextual ambiguity than autistic subjects with lower social-cognitive traits did (see Fig. 9).

### Supplementary robustness analyses

Given group differences in depressive symptoms and ADHD-related traits, we conducted additional supplementary GLMMs for all significant interaction effects, including either BDI or WURSK sum scores as covariates. Several interaction effects remained statistically significant, whereas others were attenuated and did not reach significance in all models. Full results are reported in the Supplementary Material (Section 5).

## Discussion

We investigated FER performance in autistic and non-autistic adults, focusing on task adaptation and stimulus salience. Consistent with prior research, ASD participants showed lower accuracy and longer RTs [7]. In addition to group differences, mixed-effects modeling and automated FER revealed meaningful participant and item-level variance.

At the participant level, we found evidence of task adaptation over time, with stronger adaptation among non-autistic participants. At the stimulus level, we found that target-distractor differences moderated group performance such that NC benefited more from higher probability differences than ASD. Self-reported social responsiveness predicted FER ability within both groups, and self-reported social-cognitive traits moderated the effects of contextual ambiguity in individuals with ASD.

### Task adaptation

In contrast to our hypothesis, we found higher levels of task adaptation in the NC group than in the ASD group. While we expected to see trial-dependent variability in ASD, we expected the NC group to demonstrate consistent FER performance over time. This expectation was based on the assumption that FER is a highly trained ability in non-autistic individuals that requires a quick and reliable activation in everyday situations. However, the BERT2 is a lab-based task lacking real-world social context and time pressure. This artificial and explicit nature of the BERT2 may hinder the immediate activation of fast, implicit processing, offering room for improvement by utilizing the implicit route. Alternatively, improvements could also be achieved by reinforcing the explicit route. In this case, intensive conscious processing could increase the accuracy of the classification. The RT results may help to distinguish between the two possible explanations: while existing literature on face processing suggests a decrease in RT over time [33], we observed an overall increase in RT throughout the task. This unexpected finding supports the idea of explicit route reinforcement, as increases in RT may indicate a strategic change in speed-accuracy trade-off.

The finding of decreased task adaptation in ASD might be related to reduced cognitive flexibility [34], which could limit adjustments in processing strategies over the course of the task. The results of the stimulus saliency analysis may indicate an underlying mechanism of task adaptation, as discussed in more detail below. Nevertheless, even without causal assumptions, our trial-based analysis could have significant implications for interpreting FER experiments in general: if FER accuracy develops differently over the course of an experiment for ASD and NC, the number of repeated measures can lead to substantial biases in the estimated FER differences in ASD. These findings support the inclusion of trial number as a covariate in mixed-effects logistic regression models as an advancement over classical statistical analyses with aggregate accuracies per participant.

### Stimulus salience

Contrary to our expectations, we did not find a direct influence of any estimate of stimulus salience on FER accuracy. However, target-probability and contextual ambiguity significantly predicted RT for both groups, indicating that FaceReader’s probability scores align with participants’ efficiency in extracting meaningful emotional information from stimulus faces. The dissociation of RT and accuracy may indicate that the estimated probabilities represent intensity rather than the prototypicality of the expressions: lower intensity expressions require more time for evidence accumulation but still yield correct responses given sufficient time. Moreover, the forced‑choice design of the BERT2, with a limited response set and relatively high overall accuracy, may constrain the extent to which stimulus salience manifests as accuracy differences.

The lack of interaction between target-probability and autism diagnosis aligns with meta-analytic evidence [7], suggesting that emotion intensity does not account for autism-related difficulties in FER. The fact that overall ambiguity had no influence on accuracy or RT also suggests that the subjects did not consider all six basic emotions when classifying facial expressions in the BERT2. Importantly, contextual ambiguity moderated group differences. Contrary to our hypothesis, the results suggest larger group differences for stimuli with low contextual ambiguity, suggesting that NCs were less affected by near-neighbor distractors.

While our experimental design does not allow direct inferences on strategy use, this pattern is consistent with the possibility that NCs did not (exclusively) rely on direct emotion labeling, but rather inferred the correct emotion label based on the response options provided. This “process of elimination” has previously been proposed as a compensatory mechanism for autistic individuals to mask deficits in FER [35]. Ironically however, our results suggest the opposite pattern: NCs show a stronger tendency to exclude response options or at least appear to be more capable of doing so. Using response options as a cue might seem to be an artificial workaround for ‘real’ emotion recognition. However, response options can be seen as contextual information, and the significant interaction effect of contextual ambiguity and social-cognitive traits on FER accuracy in autistic individuals may suggest that the use of this contextual information might indeed rely on a social-cognitive process.

Zalla & Korman (2018) formulated the “integration deficit hypothesis” to explain why high-functioning autistic adults commonly pass basic theory of mind (ToM) tasks such as first- and second-order false belief tasks but demonstrate pervasive difficulties in advanced ToM tasks such as the Faux Pas and the Strange Stories tasks [36]. According to their hypothesis, high-functioning autistic adults can use rule-based cognitive strategies to solve false belief tasks. However, they struggle to efficiently integrate information about mental states with knowledge about social situations or the broader behavioral context, as required in advanced ToM tasks. Similarly, sensitive FER tasks such as BERT2 may require the integration of emotional information from faces and contextual information from response options to achieve high performance levels. This interpretation is also consistent with predictive coding accounts of autism, which posit that autistic individuals have less flexible and more context-independent priors in belief updating [37].

### Robustness of effects to co-occurring symptoms

Because depressive symptoms and ADHD-related traits were elevated in the ASD group, we conducted supplementary robustness analyses to assess their potential contribution to the observed interaction effects. While some interactions were attenuated after accounting for these covariates, others—most notably the interaction between diagnostic group and contextual ambiguity—remained robust. This pattern suggests that autism-related characteristics play a central role in the reported effects, while co-occurring symptom dimensions may partially modulate specific aspects of task performance (see Supplementary Material, Section 5).

### Using automated FER for autism research

We used FaceReader 9, a computer vision software that uses machine learning to detect emotional expressions in faces, to inform our analysis of autistic peculiarities in FER. While machine learning approaches using computer vision have already been shown to be useful in predicting autism diagnoses on the basis of facial expressions during social interactions [38], this is the first study utilizing an automated FER model as a reference for human FER in autism research.

A comparison of FaceReader’s classification performance with the experimental data shows that it does not outperform non-autistic participants but achieves a similar level of performance. The FaceReader results therefore cannot be considered an “objective” measure of the emotional information displayed by the faces. Despite performing similarly to NC, FaceReader values cannot be regarded as a neurotypical benchmark either, as the error profile does not reflect the participants’ difficulties (see Supplementary Table S2.2 for comparison).

Nevertheless, we argue that automated FER models are useful for understanding differences between autistic and non-autistic individuals in FER, as they provide a sample-independent and paradigm-independent probability space across all emotions. Furthermore, the significant correlations between FaceReader parameters and behavioral data, particularly RT, demonstrate that the automated FER model captured psychologically relevant information for human FER.

### General discussion

Our results demonstrate that mixed-effects modeling provides valuable insights into the mechanisms that make up aggregate FER accuracy scores by accounting for item and participant variance. Unraveling these mechanisms may be crucial for understanding the difficulties of autistic individuals in FER.

The inclusion of trial number as a predictor revealed greater task adaptation and increased RT, suggesting that both groups adapted to the task demands by utilizing additional resources, trading off speed for accuracy. However, the significant interaction of group and trial number on accuracies additionally indicates that NCs benefited more from this adaptation than autistic participants. Furthermore, the inclusion of automated FER model parameters revealed that NCs were more sensitive to differences in the composition of response options. This, in turn, could indicate that the additional resources leading to improved task adaptation are based on increased consideration of the response options. The results thus contradict the assumption that autistic individuals rely more heavily on cognitive strategies during FER than non-autistic individuals do. On the contrary, NCs appear to be able to exploit specific task characteristics in the course of the experiment to improve their performance. This demonstrates a critical issue of FER research in ASD: the various experimental tests used to assess FER performance may measure different abilities, depending on the specific task design, including factors such as test duration, response options, and stimulus composition.

Investigating these influences on the characteristics of FER in ASD using mixed-effects modeling could help design more reliable and cognitively transparent tests. Moreover, it could also improve our understanding of the large body of literature on FER in autism, as the methods described here can easily be applied to post-hoc analyses of past studies.

### Limitations

Several potential limitations of the present study should be acknowledged. First, our conclusions are specific to our task design and sample composition. The effects of task adaptation and stimulus salience could differ for other FER tasks. Second, the relatively long testing session may have influenced the participants’ performance, although the increase in accuracy over time suggests that fatigue was not a major factor. Third, the restriction of our sample to autistic adults without intellectual disabilities means that no conclusions can be drawn for the entire autism spectrum. Furthermore, we cannot draw direct conclusions about the attentional processes during the task. Future studies could examine relative attention to faces and response labels via eye-tracking to test our exclusion hypothesis.

Another limitation is our singular focus on FER, while emotion recognition is multimodal [3] and difficulties in ASD extend to other modalities, such as voices [7, 39]. Future studies may explore whether the observed effects generalize across modalities.

Caution is also warranted when interpreting automated FER estimates. While automated FER models provide fast and easy-to-adapt estimates of classification probability, the underlying decision-making processes remain opaque [40]. Our interpretations of stimulus salience parameters as measures of emotion ambiguity or intensity are based on theoretical assumptions and do not follow directly from the model. While our study provides some empirical evidence for the feasibility of these estimates, future research may directly assess facial action coding [21] to reliably disambiguate the effects of emotion prototypicality, ambiguity or intensity.

Furthermore, FaceReader 9’s classification accuracy on BERT2 is lower than that on standardized datasets such as the Radboud Faces Database, which additionally complicates the interpretation of stimulus parameters. While this decrease in accuracy for less standardized faces has already been observed [23], it could be attributed either to the FER model or to the stimulus material. Developments in machine learning could improve classification accuracy in future models. Additionally, systematically comparing the classification errors of automated FER models with human errors could enhance our understanding of these models’ strengths and weaknesses.

One of the key strengths of our study is its relatively large sample size. Moreover, as a satellite project closely linked to the largest controlled, randomized, multicenter psychotherapy trial to date in adults with ASD [24]—meeting all the rigorous standards of a phase III trial—our study benefits from clinical and psychometric assessments conducted at the highest level of quality.

## Conclusions

This study provides novel insights into the mechanisms underlying FER differences in autistic adults by employing mixed-effects modeling to investigate trial- and stimulus-level interactions with autism diagnosis. Our results indicate that autistic individuals exhibit reduced task adaptation in FER, suggesting challenges in flexibly adjusting to repeated emotion recognition demands. Notably, contextual ambiguity influenced performance differently across groups, with non-autistic participants benefiting more from informative response options—a finding that challenges assumptions about autistic reliance on compensatory strategies. Within the autistic group, social-cognitive traits moderated the impact of contextual cues, underscoring the heterogeneity of FER abilities in autism and emphasizing the importance of integrating contextual information in social settings.

The integration of automated FER measures with mixed-effects modeling allowed us to disentangle item-level and participant-level contributions to FER performance. This approach not only enhances our understanding of social-cognitive processing in individuals with autism but also highlights the limitations of focusing solely on aggregate accuracy scores. Future studies on FER in individuals with autism could profit from these insights, for example, by controlling for trial number or systematically manipulating the amount of contextual information provided by the task design. The analyses presented here could also prove fruitful for the post-hoc examination of existing experimental data on FER in ASD and may be adapted for meta-analyses investigating the influence of the identified factors on a larger scale.

## Electronic supplementary material

Below is the link to the electronic supplementary material.

Supplementary Material 1

Supplementary Material 2

## Data Availability

The datasets generated and/or analyzed during the current study are available in the Open Science Framework, https://doi.org/10.17605/OSF.IO/BW29C. The BERT2 is available online under GNU General Public License 3.0 from https://doi.org/10.18452/20019.

## Abbreviations

ADHD: Attention-Deficit/Hyperactivity Disorder
ADOS-2: Autism Diagnostic Observation Schedule 2
ASD: Autism Spectrum Disorder
BDI: Beck Depression Inventory
BERT: Berlin Emotion Recognition Test
FER: Facial Emotion Recognition
GLMM: Generalized Linear Mixed-Effects Model
ICD-10: International Classification of Diseases, 10th revision
IQ: Intelligence Quotient
logRT: Log-Transformed Response Time
MWT-B: Mehrfachwahl-Wortschatz-Intelligenztest, Version B
NC: Non-Autistic Comparison
SRS-2: Social Responsiveness Scale 2
ToM: Theory of Mind
WURS-K: Wender Utah Rating Scale, short form
