# Supplementary material for: Reduced task adaptation and contextual awareness in autistic adults during facial emotion recognition: evidence from mixed-effects modeling and automated facial analysis
Source: Mol Autism. 2026 Mar 11;17:16. doi: 10.1186/s13229-026-00711-6 (PMC12983792; doi:10.1186/s13229-026-00711-6)
Supplement: Supplementary file 2 — Supplementary Material 2 [file 13229_2026_711_MOESM2_ESM.html]

Supplementary Materials: Reduced Task Adaptation and Contextual Awareness in Autistic Adults during Facial Emotion Recognition


Code 

- Show All Code
- Hide All Code

# Supplementary Materials: Reduced Task Adaptation and Contextual Awareness in Autistic Adults during Facial Emotion Recognition

#### Simon Kirsch, Hanna Drimalla, William Saakyan, Bastian Sajonz, Justus Gritzmann, Simon Maier, Thomas Fangmeier, Muyu Lin, Simón Guendelman, Christian Kaufmann, Isabel Dziobek, Ludger Tebartz van Elst

#### 2025-09-18

MIT LICENCE

The software is provided "as is", without warranty of any kind, express or implied, including but not limited to the warranties of merchantability, fitness for a particular purpose and noninfringement. In no event shall the authors or copyright holders be liable for any claim, damages or other liability, whether in an action of contract, tort or otherwise, arising from, out of or in connection with the software or the use or other dealings in the software.

Versions and citations of all packages used are listed in the reference section of this file.

# 1 Summary of Behavioral and FaceReader results

## 1.1 Stimulus properties

```
table.s1 <- stim_table %>%
  kbl(caption = 'Stimulus Properties. Each stimulus face was presented with three response options: one target label and two distractor labels. Label positions were randomized across stimuli. /n emo_label = target emotion label; target_prob = target probability; delta = target-distractor difference', digits = 3) %>%
  kable_styling(bootstrap_options = c("striped", "hover", "condensed", "responsive",
                                      full_width = F, position = "float_left"))

add_header_above(table.s1, c(" " = 6, "FaceReader Probabilities" = 6,
                             "Stimulus Parameters" = 3))
```

Table 1.1: Stimulus Properties. Each stimulus face was presented with three response options: one target label and two distractor labels. Label positions were randomized across stimuli. /n emo\_label = target emotion label; target\_prob = target probability; delta = target-distractor difference

|  | | | | | | FaceReader Probabilities | | | | | | Stimulus Parameters | | |
| --- | --- | --- | --- | --- | --- | --- | --- | --- | --- | --- | --- | --- | --- | --- |
| pic | actor | gender\_actor | emo\_label | distractor | distractor2 | happieness | sadness | anger | surprise | fear | disgust | target\_prob | entropy | delta |
| angry\_EmGumz\_25\_plus4.jpg | Gumz | m | anger | fear | happieness | 0.000 | 0.004 | 0.367 | 0.054 | 0.015 | 0.002 | 0.367 | 0.896 | 0.352 |
| angry\_EmKocevski\_25.jpg | Kocevski | m | anger | disgust | happieness | 0.000 | 0.003 | 0.934 | 0.010 | 0.000 | 0.003 | 0.934 | 0.214 | 0.931 |
| angry\_EmLevent.jpg | Levent | m | anger | fear | happieness | 0.000 | 0.006 | 0.992 | 0.013 | 0.000 | 0.001 | 0.992 | 0.142 | 0.992 |
| angry\_EmSchufatinski\_50.jpg | Schufatinski | m | anger | fear | disgust | 0.000 | 0.000 | 0.228 | 0.000 | 0.021 | 0.200 | 0.228 | 1.068 | 0.028 |
| angry\_EwJung\_50.jpg | Jung | f | anger | fear | disgust | 0.000 | 0.000 | 0.137 | 0.000 | 0.000 | 0.000 | 0.137 | 0.397 | 0.137 |
| angry\_EwMicas\_75.jpg | Micas | f | anger | sadness | happieness | 0.001 | 0.066 | 0.132 | 0.033 | 0.006 | 0.008 | 0.132 | 0.912 | 0.066 |
| angry\_EwPerlick\_50.jpg | Perlick | f | anger | surprise | fear | 0.000 | 0.057 | 0.013 | 0.007 | 0.174 | 0.000 | 0.013 | 0.810 | -0.161 |
| angry\_EwRothe\_50.jpg | Rothe | f | anger | fear | disgust | 0.000 | 0.005 | 0.690 | 0.000 | 0.054 | 0.000 | 0.690 | 0.639 | 0.635 |
| anxious\_EmPatzke\_75.jpg | Patzke | m | fear | surprise | sadness | 0.000 | 0.000 | 0.000 | 0.603 | 0.257 | 0.000 | 0.257 | 0.946 | -0.346 |
| anxious\_EmSchufatinski\_50.jpg | Schufatinski | m | fear | sadness | disgust | 0.000 | 0.000 | 0.001 | 0.311 | 0.406 | 0.000 | 0.406 | 1.066 | 0.406 |
| anxious\_EwBerg.jpg | Berg | f | fear | anger | happieness | 0.000 | 0.000 | 0.000 | 0.060 | 0.980 | 0.000 | 0.980 | 0.276 | 0.980 |
| anxious\_EwMicas\_75.jpg | Micas | f | fear | sadness | disgust | 0.000 | 0.000 | 0.000 | 0.860 | 0.323 | 0.000 | 0.323 | 0.719 | 0.323 |
| disgust\_EmFoerster\_75.jpg | Foerster | m | disgust | anger | happieness | 0.000 | 0.004 | 0.007 | 0.000 | 0.000 | 0.648 | 0.648 | 0.487 | 0.642 |
| disgust\_EmKocevski\_75.jpg | Kocevski | m | disgust | anger | sadness | 0.000 | 0.013 | 0.004 | 0.000 | 0.000 | 0.995 | 0.995 | 0.123 | 0.982 |
| disgust\_EmWalter\_50.jpg | Walter | m | disgust | anger | surprise | 0.015 | 0.060 | 0.000 | 0.000 | 0.000 | 0.167 | 0.167 | 0.771 | 0.167 |
| disgust\_EmWeststroem.jpg | Westroem | m | disgust | anger | sadness | 0.000 | 0.000 | 0.000 | 0.000 | 0.000 | 0.987 | 0.987 | 0.024 | 0.987 |
| disgust\_EwGospadar\_75.jpg | Gospadar | f | disgust | sadness | surprise | 0.000 | 0.000 | 0.001 | 0.000 | 0.000 | 0.996 | 0.996 | 0.017 | 0.996 |
| disgust\_EwHuebner\_100.jpg | Huebner | f | disgust | anger | surprise | 0.000 | 0.000 | 0.005 | 0.000 | 0.000 | 0.996 | 0.996 | 0.045 | 0.991 |
| disgust\_EwOtt\_75.jpg | Ott | f | disgust | sadness | happieness | 0.002 | 0.026 | 0.003 | 0.000 | 0.007 | 0.587 | 0.587 | 0.679 | 0.561 |
| disgust\_EwSmuda.jpg | Smuda | f | disgust | anger | sadness | 0.000 | 0.024 | 0.000 | 0.000 | 0.000 | 0.908 | 0.908 | 0.261 | 0.883 |
| joyful\_EmFoerster\_12.jpg | Foerster | m | happieness | sadness | disgust | 0.089 | 0.002 | 0.000 | 0.009 | 0.001 | 0.000 | 0.089 | 0.400 | 0.087 |
| joyful\_EmKocevski\_12.jpg | Kocevski | m | happieness | sadness | anger | 0.009 | 0.015 | 0.001 | 0.023 | 0.000 | 0.001 | 0.009 | 0.295 | -0.005 |
| joyful\_EmLevent\_25.jpg | Levent | m | happieness | sadness | surprise | 0.262 | 0.003 | 0.005 | 0.038 | 0.000 | 0.000 | 0.262 | 0.748 | 0.224 |
| joyful\_EmRothkirch\_37.jpg | Rothkirch | m | happieness | anger | surprise | 0.000 | 0.023 | 0.000 | 0.005 | 0.000 | 0.000 | 0.000 | 0.165 | -0.004 |
| joyful\_EwBraun\_37.jpg | Braun | f | happieness | sadness | fear | 0.119 | 0.090 | 0.000 | 0.009 | 0.063 | 0.002 | 0.119 | 1.005 | 0.029 |
| joyful\_EwFriedrich\_12.jpg | Friedrich | f | happieness | surprise | disgust | 0.003 | 0.001 | 0.000 | 0.662 | 0.000 | 0.000 | 0.003 | 0.429 | -0.659 |
| joyful\_EwNeukirchner.jpg | Neukirchner | f | happieness | surprise | fear | 0.051 | 0.000 | 0.000 | 0.007 | 0.000 | 0.000 | 0.051 | 0.274 | 0.044 |
| joyful\_EwStudelny\_50.jpg | Studelny | f | happieness | surprise | anger | 0.003 | 0.001 | 0.001 | 0.191 | 0.044 | 0.000 | 0.003 | 0.696 | -0.189 |
| sad\_EmGumz\_25.jpg | Gumz | m | sadness | disgust | fear | 0.003 | 0.455 | 0.003 | 0.002 | 0.000 | 0.010 | 0.455 | 0.650 | 0.445 |
| sad\_EmKocevski\_25.jpg | Kocevski | m | sadness | fear | disgust | 0.000 | 0.516 | 0.006 | 0.046 | 0.005 | 0.000 | 0.516 | 0.779 | 0.511 |
| sad\_EmLexow\_37.jpg | Lexow | m | sadness | anger | surprise | 0.000 | 0.025 | 0.008 | 0.003 | 0.001 | 0.001 | 0.025 | 0.224 | 0.017 |
| sad\_EmRothkirch\_25.jpg | Rothkirch | m | sadness | fear | disgust | 0.000 | 0.989 | 0.002 | 0.000 | 0.000 | 0.000 | 0.989 | 0.034 | 0.989 |
| sad\_EwBerg.jpg | Berg | f | sadness | fear | disgust | 0.000 | 0.365 | 0.001 | 0.040 | 0.000 | 0.000 | 0.365 | 0.724 | 0.365 |
| sad\_EwGospodar\_37.jpg | Gospodar | f | sadness | disgust | fear | 0.008 | 0.602 | 0.002 | 0.002 | 0.000 | 0.001 | 0.602 | 0.545 | 0.601 |
| sad\_EwMicas\_75.jpg | Micas | f | sadness | anger | surprise | 0.000 | 0.622 | 0.000 | 0.158 | 0.001 | 0.000 | 0.622 | 0.865 | 0.463 |
| sad\_EwStudelny\_25.jpg | Studelny | f | sadness | anger | disgust | 0.000 | 0.649 | 0.001 | 0.018 | 0.001 | 0.001 | 0.649 | 0.542 | 0.648 |
| surprise\_EwSmuda.jpg | Smuda | f | surprise | happieness | fear | 0.000 | 0.000 | 0.000 | 0.762 | 0.143 | 0.000 | 0.762 | 0.706 | 0.619 |
| surprised\_EmFoerster\_75.jpg | Foerster | m | surprise | happieness | disgust | 0.000 | 0.000 | 0.001 | 0.601 | 0.229 | 0.000 | 0.601 | 0.937 | 0.601 |
| surprised\_EmGumz\_37.jpg | Gumz | m | surprise | happieness | sadness | 0.001 | 0.002 | 0.001 | 0.121 | 0.046 | 0.000 | 0.121 | 0.612 | 0.119 |
| surprised\_EmKocevski\_37.jpg | Kocevski | m | surprise | fear | happieness | 0.004 | 0.000 | 0.001 | 0.939 | 0.086 | 0.000 | 0.939 | 0.427 | 0.853 |
| surprised\_EmLexow\_12.jpg | Lexow | m | surprise | happieness | anger | 0.000 | 0.000 | 0.005 | 0.440 | 0.242 | 0.000 | 0.440 | 1.064 | 0.435 |
| surprised\_EwBliefert\_50.jpg | Bliefert | f | surprise | happieness | anger | 0.000 | 0.000 | 0.000 | 0.995 | 0.012 | 0.000 | 0.995 | 0.086 | 0.995 |
| surprised\_EwHofmann\_50.jpg | Hofmann | f | surprise | disgust | happieness | 0.001 | 0.000 | 0.000 | 0.960 | 0.038 | 0.000 | 0.960 | 0.253 | 0.958 |
| surprised\_EwPerlick\_25.jpg | Perlick | f | surprise | fear | happieness | 0.000 | 0.001 | 0.000 | 0.379 | 0.148 | 0.000 | 0.379 | 0.948 | 0.231 |

## 1.2 Comparison of FaceReader and Behavioral Results by Stimulus

```
table.s2 <- stim_results_table%>%
  kbl(caption = "Results by Stimulus. /n emo_label = target emotion label; FR = FaceReader; ASD = autism spectrum disorder; NC = non-autistic comparison", digits = 3) %>%
  kable_styling(bootstrap_options = c("striped", "hover", "condensed", "responsive",
                                      full_width = F, position = "float_left"))

add_header_above(table.s2, c(" " = 4, "FaceReader Results" = 3,
                             "Behavioral Results" = 8))
```

Table 1.2: Results by Stimulus. /n emo\_label = target emotion label; FR = FaceReader; ASD = autism spectrum disorder; NC = non-autistic comparison

|  | | | | FaceReader Results | | | Behavioral Results | | | | | | | |
| --- | --- | --- | --- | --- | --- | --- | --- | --- | --- | --- | --- | --- | --- | --- |
| pic | emo\_label | distractor | distractor2 | FR\_Classification | FR\_Correct | FR\_Correct\_adjusted | correct\_NC | dis1\_resp\_NC | dis2\_resp\_NC | RT\_NC | correct\_ASD | distractor1\_response\_ASD | distractor2\_response\_ASD | RT\_ASD |
| angry\_EmGumz\_25\_plus4.jpg | anger | fear | happieness | anger | 1 | 1 | 0.784 | 0.180 | 0.036 | 3334.730 | 0.675 | 0.250 | 0.075 | 4516.950 |
| angry\_EmKocevski\_25.jpg | anger | disgust | happieness | anger | 1 | 1 | 0.910 | 0.063 | 0.027 | 2791.270 | 0.858 | 0.100 | 0.042 | 3708.192 |
| angry\_EmLevent.jpg | anger | fear | happieness | anger | 1 | 1 | 0.928 | 0.036 | 0.036 | 2670.180 | 0.900 | 0.092 | 0.008 | 3592.833 |
| angry\_EmSchufatinski\_50.jpg | anger | fear | disgust | anger | 1 | 1 | 0.703 | 0.063 | 0.234 | 2861.838 | 0.725 | 0.092 | 0.183 | 3447.075 |
| angry\_EwJung\_50.jpg | anger | fear | disgust | anger | 1 | 1 | 0.910 | 0.045 | 0.045 | 3446.486 | 0.733 | 0.125 | 0.142 | 4506.533 |
| angry\_EwMicas\_75.jpg | anger | sadness | happieness | anger | 1 | 1 | 0.973 | 0.027 | 0.000 | 2418.315 | 0.892 | 0.100 | 0.008 | 3070.292 |
| angry\_EwPerlick\_50.jpg | anger | surprise | fear | fear | 0 | 0 | 0.811 | 0.099 | 0.090 | 3499.261 | 0.683 | 0.142 | 0.175 | 4613.025 |
| angry\_EwRothe\_50.jpg | anger | fear | disgust | anger | 1 | 1 | 0.676 | 0.261 | 0.063 | 3323.973 | 0.575 | 0.333 | 0.092 | 4069.767 |
| anxious\_EmPatzke\_75.jpg | fear | surprise | sadness | surprise | 0 | 0 | 0.618 | 0.364 | 0.018 | 2839.518 | 0.658 | 0.325 | 0.017 | 3497.750 |
| anxious\_EmSchufatinski\_50.jpg | fear | sadness | disgust | fear | 1 | 1 | 0.901 | 0.027 | 0.072 | 3069.171 | 0.875 | 0.042 | 0.083 | 3926.408 |
| anxious\_EwBerg.jpg | fear | anger | happieness | fear | 1 | 1 | 0.874 | 0.126 | 0.000 | 3081.883 | 0.800 | 0.200 | 0.000 | 3848.125 |
| anxious\_EwMicas\_75.jpg | fear | sadness | disgust | surprise | 0 | 1 | 0.928 | 0.045 | 0.027 | 3490.505 | 0.892 | 0.025 | 0.083 | 4311.208 |
| disgust\_EmFoerster\_75.jpg | disgust | anger | happieness | disgust | 1 | 1 | 0.856 | 0.144 | 0.000 | 2412.532 | 0.717 | 0.275 | 0.008 | 3003.025 |
| disgust\_EmKocevski\_75.jpg | disgust | anger | sadness | disgust | 1 | 1 | 0.712 | 0.216 | 0.072 | 2561.820 | 0.600 | 0.308 | 0.092 | 3125.300 |
| disgust\_EmWalter\_50.jpg | disgust | anger | surprise | disgust | 1 | 1 | 0.586 | 0.342 | 0.072 | 3338.658 | 0.608 | 0.325 | 0.067 | 4096.117 |
| disgust\_EmWeststroem.jpg | disgust | anger | sadness | disgust | 1 | 1 | 0.937 | 0.054 | 0.009 | 2407.063 | 0.800 | 0.150 | 0.050 | 2954.667 |
| disgust\_EwGospadar\_75.jpg | disgust | sadness | surprise | disgust | 1 | 1 | 0.982 | 0.018 | 0.000 | 1954.748 | 0.883 | 0.092 | 0.025 | 2612.767 |
| disgust\_EwHuebner\_100.jpg | disgust | anger | surprise | disgust | 1 | 1 | 0.748 | 0.243 | 0.009 | 2380.757 | 0.692 | 0.292 | 0.017 | 2426.625 |
| disgust\_EwOtt\_75.jpg | disgust | sadness | happieness | disgust | 1 | 1 | 0.865 | 0.126 | 0.009 | 2654.360 | 0.825 | 0.167 | 0.008 | 3685.992 |
| disgust\_EwSmuda.jpg | disgust | anger | sadness | disgust | 1 | 1 | 0.766 | 0.144 | 0.090 | 2611.441 | 0.642 | 0.233 | 0.125 | 3203.042 |
| joyful\_EmFoerster\_12.jpg | happieness | sadness | disgust | happieness | 1 | 1 | 0.937 | 0.054 | 0.009 | 3058.216 | 0.850 | 0.117 | 0.033 | 4123.342 |
| joyful\_EmKocevski\_12.jpg | happieness | sadness | anger | surprise | 0 | 0 | 0.820 | 0.162 | 0.018 | 2938.730 | 0.742 | 0.167 | 0.092 | 4311.758 |
| joyful\_EmLevent\_25.jpg | happieness | sadness | surprise | happieness | 1 | 1 | 0.964 | 0.027 | 0.009 | 2419.685 | 0.925 | 0.025 | 0.050 | 3019.800 |
| joyful\_EmRothkirch\_37.jpg | happieness | anger | surprise | sadness | 0 | 0 | 0.883 | 0.045 | 0.072 | 3364.468 | 0.758 | 0.108 | 0.133 | 4897.983 |
| joyful\_EwBraun\_37.jpg | happieness | sadness | fear | happieness | 1 | 1 | 0.784 | 0.207 | 0.009 | 3005.414 | 0.667 | 0.300 | 0.033 | 3978.242 |
| joyful\_EwFriedrich\_12.jpg | happieness | surprise | disgust | surprise | 0 | 0 | 0.847 | 0.126 | 0.027 | 3352.225 | 0.808 | 0.125 | 0.067 | 4509.792 |
| joyful\_EwNeukirchner.jpg | happieness | surprise | fear | happieness | 1 | 1 | 0.865 | 0.081 | 0.054 | 3340.586 | 0.842 | 0.125 | 0.033 | 3886.767 |
| joyful\_EwStudelny\_50.jpg | happieness | surprise | anger | surprise | 0 | 0 | 0.937 | 0.063 | 0.000 | 2148.045 | 0.883 | 0.117 | 0.000 | 2770.892 |
| sad\_EmGumz\_25.jpg | sadness | disgust | fear | sadness | 1 | 1 | 0.820 | 0.108 | 0.072 | 3220.126 | 0.708 | 0.158 | 0.133 | 4498.350 |
| sad\_EmKocevski\_25.jpg | sadness | fear | disgust | sadness | 1 | 1 | 0.919 | 0.045 | 0.036 | 2911.649 | 0.767 | 0.158 | 0.075 | 3453.083 |
| sad\_EmLexow\_37.jpg | sadness | anger | surprise | sadness | 1 | 1 | 0.829 | 0.153 | 0.018 | 2780.712 | 0.817 | 0.158 | 0.025 | 3642.767 |
| sad\_EmRothkirch\_25.jpg | sadness | fear | disgust | sadness | 1 | 1 | 0.901 | 0.072 | 0.027 | 3026.054 | 0.825 | 0.100 | 0.075 | 3845.467 |
| sad\_EwBerg.jpg | sadness | fear | disgust | sadness | 1 | 1 | 0.910 | 0.054 | 0.036 | 3078.721 | 0.817 | 0.142 | 0.042 | 3756.033 |
| sad\_EwGospodar\_37.jpg | sadness | disgust | fear | sadness | 1 | 1 | 0.910 | 0.045 | 0.045 | 2491.450 | 0.833 | 0.100 | 0.067 | 3515.708 |
| sad\_EwMicas\_75.jpg | sadness | anger | surprise | sadness | 1 | 1 | 0.820 | 0.126 | 0.054 | 3016.892 | 0.792 | 0.167 | 0.042 | 3854.142 |
| sad\_EwStudelny\_25.jpg | sadness | anger | disgust | sadness | 1 | 1 | 0.811 | 0.144 | 0.045 | 2720.144 | 0.767 | 0.158 | 0.075 | 3798.500 |
| surprise\_EwSmuda.jpg | surprise | happieness | fear | surprise | 1 | 1 | 0.874 | 0.009 | 0.117 | 2494.468 | 0.792 | 0.050 | 0.158 | 3106.900 |
| surprised\_EmFoerster\_75.jpg | surprise | happieness | disgust | surprise | 1 | 1 | 0.973 | 0.009 | 0.018 | 2448.162 | 0.883 | 0.050 | 0.067 | 3270.067 |
| surprised\_EmGumz\_37.jpg | surprise | happieness | sadness | surprise | 1 | 1 | 0.757 | 0.036 | 0.207 | 3743.928 | 0.625 | 0.033 | 0.342 | 4798.108 |
| surprised\_EmKocevski\_37.jpg | surprise | fear | happieness | surprise | 1 | 1 | 0.766 | 0.207 | 0.027 | 3494.784 | 0.700 | 0.217 | 0.083 | 3988.342 |
| surprised\_EmLexow\_12.jpg | surprise | happieness | anger | surprise | 1 | 1 | 0.892 | 0.018 | 0.090 | 3108.991 | 0.750 | 0.050 | 0.200 | 3918.775 |
| surprised\_EwBliefert\_50.jpg | surprise | happieness | anger | surprise | 1 | 1 | 0.982 | 0.000 | 0.018 | 2211.468 | 0.908 | 0.050 | 0.042 | 3071.192 |
| surprised\_EwHofmann\_50.jpg | surprise | disgust | happieness | surprise | 1 | 1 | 0.955 | 0.027 | 0.018 | 2513.144 | 0.883 | 0.042 | 0.075 | 3255.092 |
| surprised\_EwPerlick\_25.jpg | surprise | fear | happieness | surprise | 1 | 1 | 0.883 | 0.081 | 0.036 | 2985.613 | 0.825 | 0.100 | 0.075 | 3686.900 |

## 1.3 Comparison of FaceReader and Behavioral Results by Emotion

```
table.s3 <- emo_results_table%>%
  kbl(caption = 'Results by Emotion. FR = FaceReader; ASD = autism spectrum disorder; NC = non-autistic comparison', digits = 3) %>%
  kable_styling(bootstrap_options = c("striped", "hover", "condensed", "responsive",
                                      full_width = F, position = "float_left"))
table.s3
```

Table 1.3: Results by Emotion. FR = FaceReader; ASD = autism spectrum disorder; NC = non-autistic comparison

| emo\_label | FR\_Accuracy | FR\_Accuracy\_adjusted | ASD\_Accuracy | NC\_Accuracy | n\_stimuli |
| --- | --- | --- | --- | --- | --- |
| anger | 0.875 | 0.875 | 0.755 | 0.837 | 8 |
| disgust | 1.000 | 1.000 | 0.721 | 0.806 | 8 |
| fear | 0.500 | 0.750 | 0.806 | 0.830 | 4 |
| happieness | 0.500 | 0.500 | 0.809 | 0.880 | 8 |
| sadness | 1.000 | 1.000 | 0.791 | 0.865 | 8 |
| surprise | 1.000 | 1.000 | 0.796 | 0.885 | 8 |

# 2 Transforming the Response Time Variable

```
rt_dat <- fulldat %>% filter(correct == 1)

ggplot(rt_dat, aes(x = response_time)) +
  geom_histogram(aes(y = ..density..), bins = 30, fill = "skyblue", color = "black")
```

Figure 2.1: Historgram of Response Times

```
rt_dat <- rt_dat %>% mutate(logRT = log(response_time))
```

Response times are clearly non-normally distributed.To address the non-normal distribution of response times (RTs), RTs were log-transformed prior to analysis.

```
rt_dat <- rt_dat %>% mutate(logRT = log(response_time))

ggplot(rt_dat, aes(x = logRT)) +
  geom_histogram(aes(y = ..density..), bins = 30, fill = "skyblue", color = "black")
```

Figure 2.2: Historgram of log-transformed Response Times

Although log-RTs still show a slight positive skew, they are clearly more in line with a normal distribution.

# 3 Model Summaries

We present model summaries and ANOVA results for each model. Model assumptions are visually checked using the performance package.

## 3.1 Group Comparison

### 3.1.1 Accuracy

```
### Main Effect of Group
group_mod <- glmer(correct ~ Group + (1|Case) + (1|pic), data = fulldat, 
                   family = "binomial", control=glmerControl(optimizer="bobyqa"))
summary(group_mod)
```

```
## Generalized linear mixed model fit by maximum likelihood (Laplace Approximation) ['glmerMod']
##  Family: binomial  ( logit )
## Formula: correct ~ Group + (1 | Case) + (1 | pic)
##    Data: fulldat
## Control: glmerControl(optimizer = "bobyqa")
## 
##      AIC      BIC   logLik deviance df.resid 
##   9097.5   9126.4  -4544.8   9089.5    10159 
## 
## Scaled residuals: 
##     Min      1Q  Median      3Q     Max 
## -4.9770  0.2416  0.3597  0.4927  1.7739 
## 
## Random effects:
##  Groups Name        Variance Std.Dev.
##  Case   (Intercept) 0.3104   0.5571  
##  pic    (Intercept) 0.4100   0.6403  
## Number of obs: 10163, groups:  Case, 231; pic, 44
## 
## Fixed effects:
##              Estimate Std. Error z value             Pr(>|z|)    
## (Intercept)   1.70817    0.10771  15.858 < 0.0000000000000002 ***
## Group[S.ASD] -0.26766    0.04597  -5.822        0.00000000582 ***
## ---
## Signif. codes:  0 '***' 0.001 '**' 0.01 '*' 0.05 '.' 0.1 ' ' 1
## 
## Correlation of Fixed Effects:
##             (Intr)
## Grop[S.ASD] -0.041
```

```
Anova(group_mod, type = 3)
```

```
## Analysis of Deviance Table (Type III Wald chisquare tests)
## 
## Response: correct
##               Chisq Df            Pr(>Chisq)    
## (Intercept) 251.487  1 < 0.00000000000000022 ***
## Group        33.895  1        0.000000005818 ***
## ---
## Signif. codes:  0 '***' 0.001 '**' 0.01 '*' 0.05 '.' 0.1 ' ' 1
```

```
# Get Accuracy Estimate for ASD
logodds_asd <- fixef(group_mod)["(Intercept)"] + 1 * fixef(group_mod)["Group[S.ASD]"]
paste("Estimated Accuracy ASD =", round(100 *plogis(logodds_asd), 2),"%")
```

```
## [1] "Estimated Accuracy ASD = 80.85 %"
```

```
# Get Accuracy Estimate for NC
logodds_nc <- fixef(group_mod)["(Intercept)"] - 1 * fixef(group_mod)["Group[S.ASD]"]
paste("Estimated Accuracy NC =", round(100 *plogis(logodds_nc), 2),"%")
```

```
## [1] "Estimated Accuracy NC = 87.82 %"
```

```
check_model(group_mod)
```

### 3.1.2 Response Time

```
group_rt_mod <- lmer(logRT ~ Group + (1|Case) + (1|pic), data = rt_dat)
summary(group_rt_mod)
```

```
## Linear mixed model fit by REML. t-tests use Satterthwaite's method ['lmerModLmerTest']
## Formula: logRT ~ Group + (1 | Case) + (1 | pic)
##    Data: rt_dat
## 
## REML criterion at convergence: 8490
## 
## Scaled residuals: 
##     Min      1Q  Median      3Q     Max 
## -3.9864 -0.6640 -0.0620  0.6034  4.8257 
## 
## Random effects:
##  Groups   Name        Variance Std.Dev.
##  Case     (Intercept) 0.09853  0.3139  
##  pic      (Intercept) 0.02117  0.1455  
##  Residual             0.14676  0.3831  
## Number of obs: 8266, groups:  Case, 231; pic, 44
## 
## Fixed effects:
##               Estimate Std. Error        df t value             Pr(>|t|)    
## (Intercept)    7.90994    0.03044 128.95103 259.840 < 0.0000000000000002 ***
## Group[S.ASD]   0.08385    0.02110 228.42069   3.974             0.000095 ***
## ---
## Signif. codes:  0 '***' 0.001 '**' 0.01 '*' 0.05 '.' 0.1 ' ' 1
## 
## Correlation of Fixed Effects:
##             (Intr)
## Grop[S.ASD] -0.025
```

```
anova(group_rt_mod, type = 3)
```

```
## Type III Analysis of Variance Table with Satterthwaite's method
##       Sum Sq Mean Sq NumDF  DenDF F value     Pr(>F)    
## Group 2.3172  2.3172     1 228.42  15.789 0.00009496 ***
## ---
## Signif. codes:  0 '***' 0.001 '**' 0.01 '*' 0.05 '.' 0.1 ' ' 1
```

```
check_model(group_rt_mod)
```

## 3.2 Effect of Trial-Number

### 3.2.1 Accuracy

```
#### Trial Number Analysis
full_trial_mod <- glmer(correct ~ Trial_scaled * Group + (1|Case) + (1|pic), data = fulldat,                        family = "binomial")
summary(full_trial_mod)
```

```
## Generalized linear mixed model fit by maximum likelihood (Laplace Approximation) ['glmerMod']
##  Family: binomial  ( logit )
## Formula: correct ~ Trial_scaled * Group + (1 | Case) + (1 | pic)
##    Data: fulldat
## 
##      AIC      BIC   logLik deviance df.resid 
##   9066.2   9109.5  -4527.1   9054.2    10157 
## 
## Scaled residuals: 
##     Min      1Q  Median      3Q     Max 
## -5.3189  0.2352  0.3574  0.4911  1.7772 
## 
## Random effects:
##  Groups Name        Variance Std.Dev.
##  Case   (Intercept) 0.3131   0.5596  
##  pic    (Intercept) 0.4148   0.6440  
## Number of obs: 10163, groups:  Case, 231; pic, 44
## 
## Fixed effects:
##                           Estimate Std. Error z value             Pr(>|z|)    
## (Intercept)                1.71814    0.10835  15.858 < 0.0000000000000002 ***
## Trial_scaled               0.16360    0.02773   5.899        0.00000000365 ***
## Group[S.ASD]              -0.27357    0.04623  -5.917        0.00000000327 ***
## Trial_scaled:Group[S.ASD] -0.05773    0.02771  -2.083               0.0372 *  
## ---
## Signif. codes:  0 '***' 0.001 '**' 0.01 '*' 0.05 '.' 0.1 ' ' 1
## 
## Correlation of Fixed Effects:
##             (Intr) Trl_sc G[S.AS
## Trial_scald  0.029              
## Grop[S.ASD] -0.043 -0.035       
## T_:G[S.ASD] -0.015 -0.181  0.057
```

```
Anova(full_trial_mod, type = 3)
```

```
## Analysis of Deviance Table (Type III Wald chisquare tests)
## 
## Response: correct
##                       Chisq Df            Pr(>Chisq)    
## (Intercept)        251.4732  1 < 0.00000000000000022 ***
## Trial_scaled        34.8036  1        0.000000003647 ***
## Group               35.0142  1        0.000000003273 ***
## Trial_scaled:Group   4.3401  1               0.03723 *  
## ---
## Signif. codes:  0 '***' 0.001 '**' 0.01 '*' 0.05 '.' 0.1 ' ' 1
```

```
check_model(full_trial_mod)
```

#### 3.2.1.1 Post-Hoc Model: NC

```
nc_trial_mod <- glmer(correct ~ Trial_scaled + (1|Case) + (1|pic), data = nc_dat, 
                      family = "binomial", control=glmerControl(optimizer="bobyqa"))
summary(nc_trial_mod)
```

```
## Generalized linear mixed model fit by maximum likelihood (Laplace Approximation) ['glmerMod']
##  Family: binomial  ( logit )
## Formula: correct ~ Trial_scaled + (1 | Case) + (1 | pic)
##    Data: nc_dat
## Control: glmerControl(optimizer = "bobyqa")
## 
##      AIC      BIC   logLik deviance df.resid 
##     3824     3850    -1908     3816     4879 
## 
## Scaled residuals: 
##     Min      1Q  Median      3Q     Max 
## -5.4987  0.2211  0.3232  0.4346  1.2432 
## 
## Random effects:
##  Groups Name        Variance Std.Dev.
##  Case   (Intercept) 0.2093   0.4575  
##  pic    (Intercept) 0.6122   0.7824  
## Number of obs: 4883, groups:  Case, 111; pic, 44
## 
## Fixed effects:
##              Estimate Std. Error z value             Pr(>|z|)    
## (Intercept)   2.03079    0.13565  14.971 < 0.0000000000000002 ***
## Trial_scaled  0.21661    0.04291   5.048          0.000000447 ***
## ---
## Signif. codes:  0 '***' 0.001 '**' 0.01 '*' 0.05 '.' 0.1 ' ' 1
## 
## Correlation of Fixed Effects:
##             (Intr)
## Trial_scald 0.048
```

```
Anova(nc_trial_mod, type = 3)
```

```
## Analysis of Deviance Table (Type III Wald chisquare tests)
## 
## Response: correct
##                Chisq Df            Pr(>Chisq)    
## (Intercept)  224.134  1 < 0.00000000000000022 ***
## Trial_scaled  25.478  1          0.0000004475 ***
## ---
## Signif. codes:  0 '***' 0.001 '**' 0.01 '*' 0.05 '.' 0.1 ' ' 1
```

```
### Function for predicted probability by trial
predict_trial_prob <- function(trial_num, model, mean_trial, sd_trial) {
  Trial_scaled <- (trial_num - mean_trial) / sd_trial
  logodds <- fixef(model)["(Intercept)"] + Trial_scaled * fixef(model)["Trial_scaled"]
  plogis(logodds)
}

prob_first <- predict_trial_prob(min(nc_dat$Trial_scaled), nc_trial_mod, mean(nc_dat$Trial_scaled), sd(nc_dat$Trial_scaled))
prob_first <- round(prob_first * 100, 2)

prob_last <- predict_trial_prob(max(nc_dat$Trial_scaled), nc_trial_mod, mean(nc_dat$Trial_scaled), sd(nc_dat$Trial_scaled))
prob_last <- round(prob_last * 100, 2)
prob_diff <- prob_last - prob_first
prob_diff <- round(prob_diff * 100, 2)

paste("Estimated First-Trial Accuracy NC =", prob_first ,"%")
```

```
## [1] "Estimated First-Trial Accuracy NC = 84.08 %"
```

```
paste("Estimated Last-Trial Accuracy NC =", prob_last ,"%")
```

```
## [1] "Estimated Last-Trial Accuracy NC = 91.66 %"
```

```
paste("Making a difference of =", prob_diff ,"%")
```

```
## [1] "Making a difference of = 758 %"
```

```
check_model(nc_trial_mod)
```

#### 3.2.1.2 Post-Hoc Model: ASD

```
asd_trial_mod <- glmer(correct ~ Trial_scaled + (1|Case) + (1|pic), data = asd_dat, 
                      family = "binomial", control=glmerControl(optimizer="bobyqa"))
summary(asd_trial_mod)
```

```
## Generalized linear mixed model fit by maximum likelihood (Laplace Approximation) ['glmerMod']
##  Family: binomial  ( logit )
## Formula: correct ~ Trial_scaled + (1 | Case) + (1 | pic)
##    Data: asd_dat
## Control: glmerControl(optimizer = "bobyqa")
## 
##      AIC      BIC   logLik deviance df.resid 
##   5294.1   5320.4  -2643.1   5286.1     5276 
## 
## Scaled residuals: 
##     Min      1Q  Median      3Q     Max 
## -4.5670  0.2583  0.4043  0.5411  1.5966 
## 
## Random effects:
##  Groups Name        Variance Std.Dev.
##  Case   (Intercept) 0.3886   0.6234  
##  pic    (Intercept) 0.3055   0.5528  
## Number of obs: 5280, groups:  Case, 120; pic, 44
## 
## Fixed effects:
##              Estimate Std. Error z value             Pr(>|z|)    
## (Intercept)   1.42712    0.10790  13.227 < 0.0000000000000002 ***
## Trial_scaled  0.10340    0.03532   2.928              0.00342 ** 
## ---
## Signif. codes:  0 '***' 0.001 '**' 0.01 '*' 0.05 '.' 0.1 ' ' 1
## 
## Correlation of Fixed Effects:
##             (Intr)
## Trial_scald 0.019
```

```
Anova(asd_trial_mod, type = 3)
```

```
## Analysis of Deviance Table (Type III Wald chisquare tests)
## 
## Response: correct
##                 Chisq Df            Pr(>Chisq)    
## (Intercept)  174.9472  1 < 0.00000000000000022 ***
## Trial_scaled   8.5707  1              0.003416 ** 
## ---
## Signif. codes:  0 '***' 0.001 '**' 0.01 '*' 0.05 '.' 0.1 ' ' 1
```

```
prob_first <- predict_trial_prob(min(asd_dat$Trial_scaled), asd_trial_mod, mean(asd_dat$Trial_scaled), sd(asd_dat$Trial_scaled))
prob_first <- round(prob_first * 100, 2)

prob_last <- predict_trial_prob(max(asd_dat$Trial_scaled), asd_trial_mod, mean(asd_dat$Trial_scaled), sd(asd_dat$Trial_scaled))
prob_last <- round(prob_last * 100, 2)
prob_diff <- prob_last - prob_first
prob_diff <- round(prob_diff * 100, 2)

paste("Estimated First-Trial Accuracy ASD =", prob_first ,"%")
```

```
## [1] "Estimated First-Trial Accuracy ASD = 77.77 %"
```

```
paste("Estimated Last-Trial Accuracy ASD =", prob_last ,"%")
```

```
## [1] "Estimated Last-Trial Accuracy ASD = 83.23 %"
```

```
paste("Making a difference of =", prob_diff ,"%")
```

```
## [1] "Making a difference of = 546 %"
```

```
check_model(asd_trial_mod)
```

P-value adjustment:

```
p_asd <- coef(summary(asd_trial_mod))[, "Pr(>|z|)"][2]
p_nc <- coef(summary(nc_trial_mod))[, "Pr(>|z|)"][2]
p_adjusted <- p.adjust(c(p_asd, p_nc), method = "holm")

paste("Adjusted p-values: ASD =", p_adjusted[1], "NC =", p_adjusted[2])
```

```
## [1] "Adjusted p-values: ASD = 0.00341616957561628 NC = 0.000000894964339369372"
```

### 3.2.2 Response Time

```
full_trial_mod_rt <- lmer(logRT ~ Trial_scaled * Group + (1|Case) + (1|pic), data = rt_dat)
summary(full_trial_mod_rt)
```

```
## Linear mixed model fit by REML. t-tests use Satterthwaite's method ['lmerModLmerTest']
## Formula: logRT ~ Trial_scaled * Group + (1 | Case) + (1 | pic)
##    Data: rt_dat
## 
## REML criterion at convergence: 8500.3
## 
## Scaled residuals: 
##     Min      1Q  Median      3Q     Max 
## -4.0000 -0.6635 -0.0576  0.6004  4.8061 
## 
## Random effects:
##  Groups   Name        Variance Std.Dev.
##  Case     (Intercept) 0.09846  0.3138  
##  pic      (Intercept) 0.02115  0.1454  
##  Residual             0.14665  0.3830  
## Number of obs: 8266, groups:  Case, 231; pic, 44
## 
## Fixed effects:
##                              Estimate  Std. Error          df t value             Pr(>|t|)    
## (Intercept)                  7.909608    0.030428  128.984587 259.947 < 0.0000000000000002 ***
## Trial_scaled                 0.010945    0.004291 7997.340867   2.551               0.0108 *  
## Group[S.ASD]                 0.083999    0.021094  228.428705   3.982            0.0000918 ***
## Trial_scaled:Group[S.ASD]   -0.004793    0.004290 7997.145624  -1.117               0.2640    
## ---
## Signif. codes:  0 '***' 0.001 '**' 0.01 '*' 0.05 '.' 0.1 ' ' 1
## 
## Correlation of Fixed Effects:
##             (Intr) Trl_sc G[S.AS
## Trial_scald -0.004              
## Grop[S.ASD] -0.025  0.000       
## T_:G[S.ASD]  0.001  0.012 -0.005
```

```
anova(full_trial_mod_rt, type = 3)
```

```
## Type III Analysis of Variance Table with Satterthwaite's method
##                     Sum Sq Mean Sq NumDF  DenDF F value     Pr(>F)    
## Trial_scaled       0.95413 0.95413     1 7997.3  6.5061    0.01077 *  
## Group              2.32548 2.32548     1  228.4 15.8570 0.00009184 ***
## Trial_scaled:Group 0.18301 0.18301     1 7997.1  1.2479    0.26398    
## ---
## Signif. codes:  0 '***' 0.001 '**' 0.01 '*' 0.05 '.' 0.1 ' ' 1
```

```
check_model(full_trial_mod_rt)
```

#### 3.2.2.1 Post-Hoc Model: NC

```
asd_trial_rt_mod <- lmer(logRT ~ Trial_scaled + (1|Case) + (1|pic), data = asd_rt_dat)
summary(asd_trial_rt_mod)
```

```
## Linear mixed model fit by REML. t-tests use Satterthwaite's method ['lmerModLmerTest']
## Formula: logRT ~ Trial_scaled + (1 | Case) + (1 | pic)
##    Data: asd_rt_dat
## 
## REML criterion at convergence: 4704.2
## 
## Scaled residuals: 
##     Min      1Q  Median      3Q     Max 
## -3.9047 -0.6543 -0.0456  0.5943  4.4953 
## 
## Random effects:
##  Groups   Name        Variance Std.Dev.
##  Case     (Intercept) 0.13533  0.3679  
##  pic      (Intercept) 0.02399  0.1549  
##  Residual             0.16184  0.4023  
## Number of obs: 4104, groups:  Case, 120; pic, 44
## 
## Fixed effects:
##                 Estimate  Std. Error          df t value            Pr(>|t|)    
## (Intercept)     7.992811    0.041403  150.894946 193.049 <0.0000000000000002 ***
## Trial_scaled    0.005960    0.006428 3947.949536   0.927               0.354    
## ---
## Signif. codes:  0 '***' 0.001 '**' 0.01 '*' 0.05 '.' 0.1 ' ' 1
## 
## Correlation of Fixed Effects:
##             (Intr)
## Trial_scald -0.004
```

```
anova(asd_trial_rt_mod, type = 3)
```

```
## Type III Analysis of Variance Table with Satterthwaite's method
##               Sum Sq Mean Sq NumDF  DenDF F value Pr(>F)
## Trial_scaled 0.13912 0.13912     1 3947.9  0.8596 0.3539
```

```
check_model(asd_trial_rt_mod)
```

#### 3.2.2.2 Post-Hoc Model: ASD

```
nc_trial_rt_mod <- lmer(logRT ~ Trial_scaled + (1|Case) + (1|pic), data = nc_rt_dat)
summary(nc_trial_rt_mod)
```

```
## Linear mixed model fit by REML. t-tests use Satterthwaite's method ['lmerModLmerTest']
## Formula: logRT ~ Trial_scaled + (1 | Case) + (1 | pic)
##    Data: nc_rt_dat
## 
## REML criterion at convergence: 3816.2
## 
## Scaled residuals: 
##     Min      1Q  Median      3Q     Max 
## -3.7771 -0.6789 -0.0622  0.6199  4.7137 
## 
## Random effects:
##  Groups   Name        Variance Std.Dev.
##  Case     (Intercept) 0.05873  0.2423  
##  pic      (Intercept) 0.01875  0.1369  
##  Residual             0.13160  0.3628  
## Number of obs: 4162, groups:  Case, 111; pic, 44
## 
## Fixed effects:
##                 Estimate  Std. Error          df t value             Pr(>|t|)    
## (Intercept)     7.826450    0.031426  126.442768 249.040 < 0.0000000000000002 ***
## Trial_scaled    0.015971    0.005728 4015.861103   2.788              0.00533 ** 
## ---
## Signif. codes:  0 '***' 0.001 '**' 0.01 '*' 0.05 '.' 0.1 ' ' 1
## 
## Correlation of Fixed Effects:
##             (Intr)
## Trial_scald -0.006
```

```
anova(nc_trial_rt_mod, type = 3)
```

```
## Type III Analysis of Variance Table with Satterthwaite's method
##              Sum Sq Mean Sq NumDF  DenDF F value   Pr(>F)   
## Trial_scaled 1.0231  1.0231     1 4015.9  7.7741 0.005325 **
## ---
## Signif. codes:  0 '***' 0.001 '**' 0.01 '*' 0.05 '.' 0.1 ' ' 1
```

```
check_model(nc_trial_rt_mod)
```

P-value adjustment:

```
p_asd <- coef(summary(asd_trial_rt_mod))[, "Pr(>|t|)"][2]
p_nc <- coef(summary(nc_trial_rt_mod))[, "Pr(>|t|)"][2]
p_adjusted <- p.adjust(c(p_asd, p_nc), method = "holm")

paste("Adjusted p-values: ASD =", p_adjusted[1], "NC =", p_adjusted[2])
```

```
## [1] "Adjusted p-values: ASD = 0.353900346854535 NC = 0.0106502917922669"
```

## 3.3 Effect of Target-Probability

### 3.3.1 Accuracy

```
full_target_prob_mod <- glmer(correct ~ target_probability * Group + (1|Case) + (1|pic), data = fulldat, 
                              family = "binomial", control=glmerControl(optimizer="bobyqa"))
summary(full_target_prob_mod)
```

```
## Generalized linear mixed model fit by maximum likelihood (Laplace Approximation) ['glmerMod']
##  Family: binomial  ( logit )
## Formula: correct ~ target_probability * Group + (1 | Case) + (1 | pic)
##    Data: fulldat
## Control: glmerControl(optimizer = "bobyqa")
## 
##      AIC      BIC   logLik deviance df.resid 
##   9099.5   9142.8  -4543.7   9087.5    10157 
## 
## Scaled residuals: 
##     Min      1Q  Median      3Q     Max 
## -4.9883  0.2405  0.3602  0.4941  1.7533 
## 
## Random effects:
##  Groups Name        Variance Std.Dev.
##  Case   (Intercept) 0.3104   0.5571  
##  pic    (Intercept) 0.4041   0.6357  
## Number of obs: 10163, groups:  Case, 231; pic, 44
## 
## Fixed effects:
##                                 Estimate Std. Error z value             Pr(>|z|)    
## (Intercept)                      1.58778    0.17401   9.124 < 0.0000000000000002 ***
## target_probability               0.24595    0.27939   0.880             0.378700    
## Group[S.ASD]                    -0.22452    0.05862  -3.830             0.000128 ***
## target_probability:Group[S.ASD] -0.09068    0.07665  -1.183             0.236807    
## ---
## Signif. codes:  0 '***' 0.001 '**' 0.01 '*' 0.05 '.' 0.1 ' ' 1
## 
## Correlation of Fixed Effects:
##             (Intr) trgt_p G[S.AS
## trgt_prbblt -0.788              
## Grop[S.ASD] -0.040  0.030       
## t_:G[S.ASD]  0.037 -0.054 -0.620
```

```
Anova(full_target_prob_mod, type = 3)
```

```
## Analysis of Deviance Table (Type III Wald chisquare tests)
## 
## Response: correct
##                            Chisq Df            Pr(>Chisq)    
## (Intercept)              83.2562  1 < 0.00000000000000022 ***
## target_probability        0.7749  1             0.3787001    
## Group                    14.6698  1             0.0001281 ***
## target_probability:Group  1.3995  1             0.2368074    
## ---
## Signif. codes:  0 '***' 0.001 '**' 0.01 '*' 0.05 '.' 0.1 ' ' 1
```

```
check_model(full_target_prob_mod)
```

### 3.3.2 Response Time

```
full_target_prob_mod_rt <- lmer(logRT ~ target_probability * Group + (1|Case) + (1|pic), data = rt_dat)
summary(full_target_prob_mod_rt)
```

```
## Linear mixed model fit by REML. t-tests use Satterthwaite's method ['lmerModLmerTest']
## Formula: logRT ~ target_probability * Group + (1 | Case) + (1 | pic)
##    Data: rt_dat
## 
## REML criterion at convergence: 8492.9
## 
## Scaled residuals: 
##     Min      1Q  Median      3Q     Max 
## -3.9828 -0.6662 -0.0617  0.6052  4.8078 
## 
## Random effects:
##  Groups   Name        Variance Std.Dev.
##  Case     (Intercept) 0.09855  0.3139  
##  pic      (Intercept) 0.01809  0.1345  
##  Residual             0.14676  0.3831  
## Number of obs: 8266, groups:  Case, 231; pic, 44
## 
## Fixed effects:
##                                   Estimate Std. Error         df t value             Pr(>|t|)    
## (Intercept)                        7.99095    0.04086   74.33049 195.573 < 0.0000000000000002 ***
## target_probability                -0.16431    0.05779   42.06583  -2.843              0.00686 ** 
## Group[S.ASD]                       0.08786    0.02190  264.75772   4.012            0.0000783 ***
## target_probability:Group[S.ASD]   -0.00811    0.01176 7996.23649  -0.690              0.49038    
## ---
## Signif. codes:  0 '***' 0.001 '**' 0.01 '*' 0.05 '.' 0.1 ' ' 1
## 
## Correlation of Fixed Effects:
##             (Intr) trgt_p G[S.AS
## trgt_prbblt -0.698              
## Grop[S.ASD] -0.018  0.000       
## t_:G[S.ASD] -0.001  0.002 -0.267
```

```
anova(full_target_prob_mod_rt, type = 3)
```

```
## Type III Analysis of Variance Table with Satterthwaite's method
##                           Sum Sq Mean Sq NumDF  DenDF F value     Pr(>F)    
## target_probability       1.18640 1.18640     1   42.1  8.0838   0.006865 ** 
## Group                    2.36289 2.36289     1  264.8 16.1000 0.00007825 ***
## target_probability:Group 0.06982 0.06982     1 7996.2  0.4757   0.490375    
## ---
## Signif. codes:  0 '***' 0.001 '**' 0.01 '*' 0.05 '.' 0.1 ' ' 1
```

```
check_model(full_target_prob_mod_rt)
```

## 3.4 Effect of General Ambiguity (Shannon Entropy)

### 3.4.1 Accuracy

```
full_entropy_mod <- glmer(correct ~ entropy * Group + (1|Case) + (1|pic), data = fulldat, 
                              family = "binomial", control=glmerControl(optimizer="bobyqa"))
summary(full_entropy_mod)
```

```
## Generalized linear mixed model fit by maximum likelihood (Laplace Approximation) ['glmerMod']
##  Family: binomial  ( logit )
## Formula: correct ~ entropy * Group + (1 | Case) + (1 | pic)
##    Data: fulldat
## Control: glmerControl(optimizer = "bobyqa")
## 
##      AIC      BIC   logLik deviance df.resid 
##   9097.9   9141.2  -4542.9   9085.9    10157 
## 
## Scaled residuals: 
##     Min      1Q  Median      3Q     Max 
## -4.9894  0.2387  0.3583  0.4922  1.7515 
## 
## Random effects:
##  Groups Name        Variance Std.Dev.
##  Case   (Intercept) 0.3104   0.5571  
##  pic    (Intercept) 0.3994   0.6320  
## Number of obs: 10163, groups:  Case, 231; pic, 44
## 
## Fixed effects:
##                      Estimate Std. Error z value             Pr(>|z|)    
## (Intercept)           1.89629    0.19836   9.560 < 0.0000000000000002 ***
## entropy              -0.34361    0.30677  -1.120                0.263    
## Group[S.ASD]         -0.34560    0.06655  -5.193          0.000000207 ***
## entropy:Group[S.ASD]  0.13803    0.08498   1.624                0.104    
## ---
## Signif. codes:  0 '***' 0.001 '**' 0.01 '*' 0.05 '.' 0.1 ' ' 1
## 
## Correlation of Fixed Effects:
##             (Intr) entrpy G[S.AS
## entropy     -0.843              
## Grop[S.ASD] -0.057  0.044       
## en:G[S.ASD]  0.053 -0.055 -0.723
```

```
Anova(full_entropy_mod, type = 3)
```

```
## Analysis of Deviance Table (Type III Wald chisquare tests)
## 
## Response: correct
##                 Chisq Df            Pr(>Chisq)    
## (Intercept)   91.3870  1 < 0.00000000000000022 ***
## entropy        1.2546  1                0.2627    
## Group         26.9669  1           0.000000207 ***
## entropy:Group  2.6383  1                0.1043    
## ---
## Signif. codes:  0 '***' 0.001 '**' 0.01 '*' 0.05 '.' 0.1 ' ' 1
```

```
check_model(full_entropy_mod)
```

### 3.4.2 Response Time

```
full_entropy_mod_rt <- lmer(logRT ~ entropy * Group + (1|Case) + (1|pic), data = rt_dat)
summary(full_entropy_mod_rt)
```

```
## Linear mixed model fit by REML. t-tests use Satterthwaite's method ['lmerModLmerTest']
## Formula: logRT ~ entropy * Group + (1 | Case) + (1 | pic)
##    Data: rt_dat
## 
## REML criterion at convergence: 8497.5
## 
## Scaled residuals: 
##     Min      1Q  Median      3Q     Max 
## -3.9866 -0.6656 -0.0611  0.6022  4.8286 
## 
## Random effects:
##  Groups   Name        Variance Std.Dev.
##  Case     (Intercept) 0.09853  0.3139  
##  pic      (Intercept) 0.02021  0.1422  
##  Residual             0.14677  0.3831  
## Number of obs: 8266, groups:  Case, 231; pic, 44
## 
## Fixed effects:
##                         Estimate  Std. Error          df t value             Pr(>|t|)    
## (Intercept)             7.846887    0.047284   63.485626 165.953 < 0.0000000000000002 ***
## entropy                 0.116116    0.067193   42.037644   1.728             0.091312 .  
## Group[S.ASD]            0.084556    0.022220  280.684319   3.805             0.000174 ***
## entropy:Group[S.ASD]   -0.001333    0.012948 7995.363007  -0.103             0.918027    
## ---
## Signif. codes:  0 '***' 0.001 '**' 0.01 '*' 0.05 '.' 0.1 ' ' 1
## 
## Correlation of Fixed Effects:
##             (Intr) entrpy G[S.AS
## entropy     -0.772              
## Grop[S.ASD] -0.015 -0.001       
## en:G[S.ASD] -0.001  0.001 -0.313
```

```
Anova(full_entropy_mod_rt, type = 3)
```

```
## Analysis of Deviance Table (Type III Wald chisquare tests)
## 
## Response: logRT
##                    Chisq Df            Pr(>Chisq)    
## (Intercept)   27540.5025  1 < 0.00000000000000022 ***
## entropy           2.9863  1             0.0839721 .  
## Group            14.4816  1             0.0001415 ***
## entropy:Group     0.0106  1             0.9180246    
## ---
## Signif. codes:  0 '***' 0.001 '**' 0.01 '*' 0.05 '.' 0.1 ' ' 1
```

```
check_model(full_entropy_mod_rt)
```

## 3.5 Effect of Contextual Ambiguity (Target-Distractor Delta)

### 3.5.1 Accuracy

```
full_delta_mod <- glmer(correct ~ delta * Group + (1|Case) + (1|pic), data = fulldat, 
                          family = "binomial", control=glmerControl(optimizer="bobyqa"))
summary(full_delta_mod)
```

```
## Generalized linear mixed model fit by maximum likelihood (Laplace Approximation) ['glmerMod']
##  Family: binomial  ( logit )
## Formula: correct ~ delta * Group + (1 | Case) + (1 | pic)
##    Data: fulldat
## Control: glmerControl(optimizer = "bobyqa")
## 
##      AIC      BIC   logLik deviance df.resid 
##   9095.9   9139.2  -4541.9   9083.9    10157 
## 
## Scaled residuals: 
##     Min      1Q  Median      3Q     Max 
## -4.9880  0.2387  0.3614  0.4927  1.7494 
## 
## Random effects:
##  Groups Name        Variance Std.Dev.
##  Case   (Intercept) 0.3105   0.5572  
##  pic    (Intercept) 0.3976   0.6306  
## Number of obs: 10163, groups:  Case, 231; pic, 44
## 
## Fixed effects:
##                    Estimate Std. Error z value             Pr(>|z|)    
## (Intercept)         1.58014    0.14638  10.795 < 0.0000000000000002 ***
## delta               0.30301    0.23560   1.286               0.1984    
## Group[S.ASD]       -0.21409    0.05258  -4.072            0.0000467 ***
## delta:Group[S.ASD] -0.13408    0.06419  -2.089               0.0367 *  
## ---
## Signif. codes:  0 '***' 0.001 '**' 0.01 '*' 0.05 '.' 0.1 ' ' 1
## 
## Correlation of Fixed Effects:
##             (Intr) delta  G[S.AS
## delta       -0.687              
## Grop[S.ASD] -0.035  0.019       
## dl:G[S.ASD]  0.024 -0.050 -0.484
```

```
Anova(full_delta_mod, type = 3)
```

```
## Analysis of Deviance Table (Type III Wald chisquare tests)
## 
## Response: correct
##                Chisq Df            Pr(>Chisq)    
## (Intercept) 116.5242  1 < 0.00000000000000022 ***
## delta         1.6541  1               0.19841    
## Group        16.5784  1            0.00004668 ***
## delta:Group   4.3637  1               0.03671 *  
## ---
## Signif. codes:  0 '***' 0.001 '**' 0.01 '*' 0.05 '.' 0.1 ' ' 1
```

```
check_model(full_delta_mod)
```

#### 3.5.1.1 Post-Hoc Model: ASD

```
delta_mod_asd <- glmer(correct ~ delta + (1|Case) + (1|pic), data = asd_dat, 
                      family = "binomial", control=glmerControl(optimizer="bobyqa"))
summary(delta_mod_asd)
```

```
## Generalized linear mixed model fit by maximum likelihood (Laplace Approximation) ['glmerMod']
##  Family: binomial  ( logit )
## Formula: correct ~ delta + (1 | Case) + (1 | pic)
##    Data: asd_dat
## Control: glmerControl(optimizer = "bobyqa")
## 
##      AIC      BIC   logLik deviance df.resid 
##   5302.0   5328.3  -2647.0   5294.0     5276 
## 
## Scaled residuals: 
##     Min      1Q  Median      3Q     Max 
## -4.6372  0.2626  0.4077  0.5446  1.6289 
## 
## Random effects:
##  Groups Name        Variance Std.Dev.
##  Case   (Intercept) 0.3866   0.6218  
##  pic    (Intercept) 0.2990   0.5468  
## Number of obs: 5280, groups:  Case, 120; pic, 44
## 
## Fixed effects:
##             Estimate Std. Error z value            Pr(>|z|)    
## (Intercept)   1.3544     0.1406   9.637 <0.0000000000000002 ***
## delta         0.1618     0.2131   0.759               0.448    
## ---
## Signif. codes:  0 '***' 0.001 '**' 0.01 '*' 0.05 '.' 0.1 ' ' 1
## 
## Correlation of Fixed Effects:
##       (Intr)
## delta -0.648
```

```
Anova(delta_mod_asd, type = 3)
```

```
## Analysis of Deviance Table (Type III Wald chisquare tests)
## 
## Response: correct
##               Chisq Df          Pr(>Chisq)    
## (Intercept) 92.8638  1 <0.0000000000000002 ***
## delta        0.5764  1              0.4477    
## ---
## Signif. codes:  0 '***' 0.001 '**' 0.01 '*' 0.05 '.' 0.1 ' ' 1
```

```
check_model(delta_mod_asd)
```

#### 3.5.1.2 Post-Hoc Model: NC

```
delta_mod_nc <- glmer(correct ~ delta + (1|Case) + (1|pic), data = nc_dat, 
                       family = "binomial", control=glmerControl(optimizer="bobyqa"))
summary(delta_mod_nc)
```

```
## Generalized linear mixed model fit by maximum likelihood (Laplace Approximation) ['glmerMod']
##  Family: binomial  ( logit )
## Formula: correct ~ delta + (1 | Case) + (1 | pic)
##    Data: nc_dat
## Control: glmerControl(optimizer = "bobyqa")
## 
##      AIC      BIC   logLik deviance df.resid 
##   3846.6   3872.6  -1919.3   3838.6     4879 
## 
## Scaled residuals: 
##     Min      1Q  Median      3Q     Max 
## -5.1845  0.2255  0.3254  0.4373  1.1750 
## 
## Random effects:
##  Groups Name        Variance Std.Dev.
##  Case   (Intercept) 0.2063   0.4542  
##  pic    (Intercept) 0.5717   0.7561  
## Number of obs: 4883, groups:  Case, 111; pic, 44
## 
## Fixed effects:
##             Estimate Std. Error z value            Pr(>|z|)    
## (Intercept)   1.8214     0.1801  10.116 <0.0000000000000002 ***
## delta         0.4497     0.2918   1.541               0.123    
## ---
## Signif. codes:  0 '***' 0.001 '**' 0.01 '*' 0.05 '.' 0.1 ' ' 1
## 
## Correlation of Fixed Effects:
##       (Intr)
## delta -0.681
```

```
Anova(delta_mod_nc, type = 3)
```

```
## Analysis of Deviance Table (Type III Wald chisquare tests)
## 
## Response: correct
##                Chisq Df          Pr(>Chisq)    
## (Intercept) 102.3309  1 <0.0000000000000002 ***
## delta         2.3749  1              0.1233    
## ---
## Signif. codes:  0 '***' 0.001 '**' 0.01 '*' 0.05 '.' 0.1 ' ' 1
```

```
check_model(delta_mod_nc)
```

### Response Time

```
full_delta_mod_rt <- lmer(logRT ~ delta * Group + (1|Case) + (1|pic), data = rt_dat)
summary(full_delta_mod_rt)
```

```
## Linear mixed model fit by REML. t-tests use Satterthwaite's method ['lmerModLmerTest']
## Formula: logRT ~ delta * Group + (1 | Case) + (1 | pic)
##    Data: rt_dat
## 
## REML criterion at convergence: 8495
## 
## Scaled residuals: 
##     Min      1Q  Median      3Q     Max 
## -3.9834 -0.6660 -0.0610  0.6043  4.8174 
## 
## Random effects:
##  Groups   Name        Variance Std.Dev.
##  Case     (Intercept) 0.09854  0.3139  
##  pic      (Intercept) 0.01858  0.1363  
##  Residual             0.14677  0.3831  
## Number of obs: 8266, groups:  Case, 231; pic, 44
## 
## Fixed effects:
##                       Estimate  Std. Error          df t value             Pr(>|t|)    
## (Intercept)           7.965700    0.036437   87.726370 218.617 < 0.0000000000000002 ***
## delta                -0.129607    0.049802   42.091819  -2.602             0.012726 *  
## Group[S.ASD]          0.085137    0.021557  248.701002   3.949             0.000102 ***
## delta:Group[S.ASD]   -0.002993    0.010055 7996.229436  -0.298             0.765957    
## ---
## Signif. codes:  0 '***' 0.001 '**' 0.01 '*' 0.05 '.' 0.1 ' ' 1
## 
## Correlation of Fixed Effects:
##             (Intr) delta  G[S.AS
## delta       -0.588              
## Grop[S.ASD] -0.021  0.000       
## dl:G[S.ASD]  0.001  0.000 -0.204
```

```
anova(full_delta_mod_rt, type = 3)
```

```
## Type III Analysis of Variance Table with Satterthwaite's method
##              Sum Sq Mean Sq NumDF  DenDF F value    Pr(>F)    
## delta       0.99405 0.99405     1   42.1  6.7728 0.0127256 *  
## Group       2.28931 2.28931     1  248.7 15.5978 0.0001021 ***
## delta:Group 0.01301 0.01301     1 7996.2  0.0886 0.7659573    
## ---
## Signif. codes:  0 '***' 0.001 '**' 0.01 '*' 0.05 '.' 0.1 ' ' 1
```

```
check_model(full_delta_mod_rt)
```

## 3.6 Effect of SRS2 Sum Score

### 3.6.1 Accuracy

#### 3.6.1.1 ASD

```
### SRS variables need to be scaled due to conversion issues: 
# Create participant-level dataframe
participant_df <- fulldat %>%
  select(Case, Group, starts_with("SRS")) %>%
  distinct()

# Scale within group
participant_df <- participant_df %>%
  group_by(Group) %>%
  mutate(SRS_scaled = scale(SRS_sum_score),
         SRS_SocialAwareness_scaled = scale(SRS_SocialAwareness_subscale_score),
         SRS_SocialCognition_scaled = scale(SRS_SocialCognition_subscale_score),
         SRS_SocialCommunication_scaled = scale(SRS_SocialCommunication_subscale_score),
         SRS_SocialMotivation_scaled = scale(SRS_SocialMotivation_subscale_score),
         SRS_AutisticMannerism_scaled = scale(SRS_AutisticMannerism_subscale_score)) %>%
  ungroup() %>% 
  select(- contains("score"))

# Merge back into trial-level data
asd_dat <- asd_dat %>%
  left_join(participant_df, by = c("Case"))

nc_dat <- nc_dat %>%
  left_join(participant_df, by = c("Case"))

### Preparation Ended

srs_mod_asd <- glmer(correct ~ SRS_scaled + (1|Case) + (1|pic), data = asd_dat, 
                     family = "binomial", control=glmerControl(optimizer="bobyqa"))
summary(srs_mod_asd)
```

```
## Generalized linear mixed model fit by maximum likelihood (Laplace Approximation) ['glmerMod']
##  Family: binomial  ( logit )
## Formula: correct ~ SRS_scaled + (1 | Case) + (1 | pic)
##    Data: asd_dat
## Control: glmerControl(optimizer = "bobyqa")
## 
##      AIC      BIC   logLik deviance df.resid 
##   5147.9   5174.0  -2569.9   5139.9     5100 
## 
## Scaled residuals: 
##     Min      1Q  Median      3Q     Max 
## -4.3610  0.2635  0.4073  0.5460  1.6725 
## 
## Random effects:
##  Groups Name        Variance Std.Dev.
##  Case   (Intercept) 0.3449   0.5873  
##  pic    (Intercept) 0.3117   0.5583  
## Number of obs: 5104, groups:  Case, 116; pic, 44
## 
## Fixed effects:
##             Estimate Std. Error z value             Pr(>|z|)    
## (Intercept)  1.40679    0.10745  13.093 < 0.0000000000000002 ***
## SRS_scaled  -0.17992    0.06513  -2.762              0.00574 ** 
## ---
## Signif. codes:  0 '***' 0.001 '**' 0.01 '*' 0.05 '.' 0.1 ' ' 1
## 
## Correlation of Fixed Effects:
##            (Intr)
## SRS_scaled -0.015
```

```
Anova(srs_mod_asd, type = 3)
```

```
## Analysis of Deviance Table (Type III Wald chisquare tests)
## 
## Response: correct
##                Chisq Df            Pr(>Chisq)    
## (Intercept) 171.4290  1 < 0.00000000000000022 ***
## SRS_scaled    7.6307  1              0.005738 ** 
## ---
## Signif. codes:  0 '***' 0.001 '**' 0.01 '*' 0.05 '.' 0.1 ' ' 1
```

```
check_model(srs_mod_asd)
```

#### 3.6.1.2 NC

```
srs_mod_nc <- glmer(correct ~ SRS_scaled + (1|Case) + (1|pic), data = nc_dat, 
                     family = "binomial", control=glmerControl(optimizer="bobyqa"))
summary(srs_mod_nc)
```

```
## Generalized linear mixed model fit by maximum likelihood (Laplace Approximation) ['glmerMod']
##  Family: binomial  ( logit )
## Formula: correct ~ SRS_scaled + (1 | Case) + (1 | pic)
##    Data: nc_dat
## Control: glmerControl(optimizer = "bobyqa")
## 
##      AIC      BIC   logLik deviance df.resid 
##   3844.9   3870.8  -1918.4   3836.9     4879 
## 
## Scaled residuals: 
##     Min      1Q  Median      3Q     Max 
## -5.1186  0.2261  0.3255  0.4373  1.1617 
## 
## Random effects:
##  Groups Name        Variance Std.Dev.
##  Case   (Intercept) 0.1910   0.4370  
##  pic    (Intercept) 0.6045   0.7775  
## Number of obs: 4883, groups:  Case, 111; pic, 44
## 
## Fixed effects:
##             Estimate Std. Error z value            Pr(>|z|)    
## (Intercept)   2.0141     0.1342  15.009 <0.0000000000000002 ***
## SRS_scaled   -0.1223     0.0593  -2.062              0.0392 *  
## ---
## Signif. codes:  0 '***' 0.001 '**' 0.01 '*' 0.05 '.' 0.1 ' ' 1
## 
## Correlation of Fixed Effects:
##            (Intr)
## SRS_scaled -0.019
```

```
check_model(srs_mod_nc)
```

### 3.6.2 Response Time

#### 3.6.2.1 ASD

```
### SRS variables need to be scaled due to conversion issues: 
# Create participant-level dataframe
participant_df_rt <- rt_dat %>%
  select(Case, Group, starts_with("SRS")) %>%
  distinct()

# Scale within group
participant_df_rt <- participant_df_rt %>%
  group_by(Group) %>%
  mutate(SRS_scaled = scale(SRS_sum_score),
         SRS_SocialAwareness_scaled = scale(SRS_SocialAwareness_subscale_score),
         SRS_SocialCognition_scaled = scale(SRS_SocialCognition_subscale_score),
         SRS_SocialCommunication_scaled = scale(SRS_SocialCommunication_subscale_score),
         SRS_SocialMotivation_scaled = scale(SRS_SocialMotivation_subscale_score),
         SRS_AutisticMannerism_scaled = scale(SRS_AutisticMannerism_subscale_score)) %>%
  ungroup() %>% 
  select(- contains("score"))

# Merge back into trial-level data
asd_rt_dat <- asd_rt_dat %>%
  left_join(participant_df_rt, by = c("Case"))

nc_rt_dat <- nc_rt_dat %>%
  left_join(participant_df, by = c("Case"))

### Preparation Ended

srs_mod_rt_asd <- lmer(logRT ~ SRS_scaled + (1|Case) + (1|pic), data = asd_rt_dat)
summary(srs_mod_rt_asd)
```

```
## Linear mixed model fit by REML. t-tests use Satterthwaite's method ['lmerModLmerTest']
## Formula: logRT ~ SRS_scaled + (1 | Case) + (1 | pic)
##    Data: asd_rt_dat
## 
## REML criterion at convergence: 4469.3
## 
## Scaled residuals: 
##     Min      1Q  Median      3Q     Max 
## -3.9534 -0.6491 -0.0482  0.5968  4.4973 
## 
## Random effects:
##  Groups   Name        Variance Std.Dev.
##  Case     (Intercept) 0.1389   0.3727  
##  pic      (Intercept) 0.0241   0.1552  
##  Residual             0.1590   0.3988  
## Number of obs: 3954, groups:  Case, 116; pic, 44
## 
## Fixed effects:
##              Estimate Std. Error        df t value            Pr(>|t|)    
## (Intercept)   7.99699    0.04228 148.12538 189.156 <0.0000000000000002 ***
## SRS_scaled    0.03435    0.03539 114.33104   0.971               0.334    
## ---
## Signif. codes:  0 '***' 0.001 '**' 0.01 '*' 0.05 '.' 0.1 ' ' 1
## 
## Correlation of Fixed Effects:
##            (Intr)
## SRS_scaled 0.001
```

```
Anova(srs_mod_rt_asd, type = 3)
```

```
## Analysis of Deviance Table (Type III Wald chisquare tests)
## 
## Response: logRT
##                  Chisq Df          Pr(>Chisq)    
## (Intercept) 35779.9345  1 <0.0000000000000002 ***
## SRS_scaled      0.9422  1              0.3317    
## ---
## Signif. codes:  0 '***' 0.001 '**' 0.01 '*' 0.05 '.' 0.1 ' ' 1
```

```
check_model(srs_mod_rt_asd)
```

## 3.7 Effect of SRS2 Social Attention Score

### 3.7.1 Accuracy

#### 3.7.1.1 ASD

```
srs_sa_mod_asd <- glmer(correct ~ SRS_SocialAwareness_scaled + (1|Case) + (1|pic), data = asd_dat, family = "binomial", control=glmerControl(optimizer="bobyqa"))
summary(srs_sa_mod_asd)
```

```
## Generalized linear mixed model fit by maximum likelihood (Laplace Approximation) ['glmerMod']
##  Family: binomial  ( logit )
## Formula: correct ~ SRS_SocialAwareness_scaled + (1 | Case) + (1 | pic)
##    Data: asd_dat
## Control: glmerControl(optimizer = "bobyqa")
## 
##      AIC      BIC   logLik deviance df.resid 
##   5145.9   5172.0  -2568.9   5137.9     5100 
## 
## Scaled residuals: 
##     Min      1Q  Median      3Q     Max 
## -4.4102  0.2639  0.4066  0.5461  1.6540 
## 
## Random effects:
##  Groups Name        Variance Std.Dev.
##  Case   (Intercept) 0.3374   0.5808  
##  pic    (Intercept) 0.3117   0.5583  
## Number of obs: 5104, groups:  Case, 116; pic, 44
## 
## Fixed effects:
##                            Estimate Std. Error z value             Pr(>|z|)    
## (Intercept)                 1.40716    0.10714  13.134 < 0.0000000000000002 ***
## SRS_SocialAwareness_scaled -0.20307    0.06498  -3.125              0.00178 ** 
## ---
## Signif. codes:  0 '***' 0.001 '**' 0.01 '*' 0.05 '.' 0.1 ' ' 1
## 
## Correlation of Fixed Effects:
##             (Intr)
## SRS_SclAwr_ -0.019
```

```
Anova(srs_sa_mod_asd, type = 3)
```

```
## Analysis of Deviance Table (Type III Wald chisquare tests)
## 
## Response: correct
##                               Chisq Df            Pr(>Chisq)    
## (Intercept)                172.4906  1 < 0.00000000000000022 ***
## SRS_SocialAwareness_scaled   9.7677  1              0.001776 ** 
## ---
## Signif. codes:  0 '***' 0.001 '**' 0.01 '*' 0.05 '.' 0.1 ' ' 1
```

```
check_model(srs_sa_mod_asd)
```

#### 3.7.1.2 NC

```
srs_sa_mod_nc <- glmer(correct ~ SRS_SocialAwareness_scaled + (1|Case) + (1|pic), data = nc_dat, 
                        family = "binomial", control=glmerControl(optimizer="bobyqa"))
summary(srs_sa_mod_nc)
```

```
## Generalized linear mixed model fit by maximum likelihood (Laplace Approximation) ['glmerMod']
##  Family: binomial  ( logit )
## Formula: correct ~ SRS_SocialAwareness_scaled + (1 | Case) + (1 | pic)
##    Data: nc_dat
## Control: glmerControl(optimizer = "bobyqa")
## 
##      AIC      BIC   logLik deviance df.resid 
##   3847.5   3873.5  -1919.7   3839.5     4879 
## 
## Scaled residuals: 
##     Min      1Q  Median      3Q     Max 
## -5.0438  0.2262  0.3247  0.4379  1.1770 
## 
## Random effects:
##  Groups Name        Variance Std.Dev.
##  Case   (Intercept) 0.2006   0.4479  
##  pic    (Intercept) 0.6044   0.7775  
## Number of obs: 4883, groups:  Case, 111; pic, 44
## 
## Fixed effects:
##                            Estimate Std. Error z value            Pr(>|z|)    
## (Intercept)                 2.01421    0.13451  14.975 <0.0000000000000002 ***
## SRS_SocialAwareness_scaled -0.07462    0.06059  -1.232               0.218    
## ---
## Signif. codes:  0 '***' 0.001 '**' 0.01 '*' 0.05 '.' 0.1 ' ' 1
## 
## Correlation of Fixed Effects:
##             (Intr)
## SRS_SclAwr_ -0.012
```

```
Anova(srs_sa_mod_nc, type = 3)
```

```
## Analysis of Deviance Table (Type III Wald chisquare tests)
## 
## Response: correct
##                               Chisq Df          Pr(>Chisq)    
## (Intercept)                224.2489  1 <0.0000000000000002 ***
## SRS_SocialAwareness_scaled   1.5168  1              0.2181    
## ---
## Signif. codes:  0 '***' 0.001 '**' 0.01 '*' 0.05 '.' 0.1 ' ' 1
```

```
check_model(srs_sa_mod_nc)
```

### 3.7.2 Response Time

#### 3.7.2.1 ASD

```
srs_sa_mod_rt_asd <- lmer(logRT ~ SRS_SocialAwareness_scaled + (1|Case) + (1|pic), data = asd_rt_dat)
summary(srs_sa_mod_rt_asd)
```

```
## Linear mixed model fit by REML. t-tests use Satterthwaite's method ['lmerModLmerTest']
## Formula: logRT ~ SRS_SocialAwareness_scaled + (1 | Case) + (1 | pic)
##    Data: asd_rt_dat
## 
## REML criterion at convergence: 4468.8
## 
## Scaled residuals: 
##     Min      1Q  Median      3Q     Max 
## -3.9519 -0.6494 -0.0483  0.5964  4.4922 
## 
## Random effects:
##  Groups   Name        Variance Std.Dev.
##  Case     (Intercept) 0.1384   0.3720  
##  pic      (Intercept) 0.0241   0.1552  
##  Residual             0.1590   0.3988  
## Number of obs: 3954, groups:  Case, 116; pic, 44
## 
## Fixed effects:
##                             Estimate Std. Error        df t value            Pr(>|t|)    
## (Intercept)                  7.99700    0.04222 148.06852 189.397 <0.0000000000000002 ***
## SRS_SocialAwareness_scaled   0.04136    0.03530 114.05174   1.172               0.244    
## ---
## Signif. codes:  0 '***' 0.001 '**' 0.01 '*' 0.05 '.' 0.1 ' ' 1
## 
## Correlation of Fixed Effects:
##             (Intr)
## SRS_SclAwr_ 0.002
```

```
Anova(srs_sa_mod_rt_asd, type = 3)
```

```
## Analysis of Deviance Table (Type III Wald chisquare tests)
## 
## Response: logRT
##                                 Chisq Df          Pr(>Chisq)    
## (Intercept)                35871.0741  1 <0.0000000000000002 ***
## SRS_SocialAwareness_scaled     1.3731  1              0.2413    
## ---
## Signif. codes:  0 '***' 0.001 '**' 0.01 '*' 0.05 '.' 0.1 ' ' 1
```

```
check_model(srs_sa_mod_rt_asd)
```

#### 3.7.2.2 NC

```
srs_sa_mod_rt_nc <- lmer(logRT ~ SRS_SocialAwareness_scaled + (1|Case) + (1|pic), data = nc_rt_dat)
summary(srs_sa_mod_rt_nc)
```

```
## Linear mixed model fit by REML. t-tests use Satterthwaite's method ['lmerModLmerTest']
## Formula: logRT ~ SRS_SocialAwareness_scaled + (1 | Case) + (1 | pic)
##    Data: nc_rt_dat
## 
## REML criterion at convergence: 3821.1
## 
## Scaled residuals: 
##     Min      1Q  Median      3Q     Max 
## -3.7616 -0.6758 -0.0660  0.6179  4.6871 
## 
## Random effects:
##  Groups   Name        Variance Std.Dev.
##  Case     (Intercept) 0.05936  0.2436  
##  pic      (Intercept) 0.01878  0.1371  
##  Residual             0.13182  0.3631  
## Number of obs: 4162, groups:  Case, 111; pic, 44
## 
## Fixed effects:
##                               Estimate  Std. Error          df t value            Pr(>|t|)    
## (Intercept)                  7.8269351   0.0315286 126.4348941 248.249 <0.0000000000000002 ***
## SRS_SocialAwareness_scaled  -0.0001497   0.0239056 108.9332757  -0.006               0.995    
## ---
## Signif. codes:  0 '***' 0.001 '**' 0.01 '*' 0.05 '.' 0.1 ' ' 1
## 
## Correlation of Fixed Effects:
##             (Intr)
## SRS_SclAwr_ 0.000
```

```
Anova(srs_sa_mod_rt_nc, type = 3)
```

```
## Analysis of Deviance Table (Type III Wald chisquare tests)
## 
## Response: logRT
##                            Chisq Df          Pr(>Chisq)    
## (Intercept)                61627  1 <0.0000000000000002 ***
## SRS_SocialAwareness_scaled     0  1               0.995    
## ---
## Signif. codes:  0 '***' 0.001 '**' 0.01 '*' 0.05 '.' 0.1 ' ' 1
```

```
check_model(srs_sa_mod_rt_nc)
```

## 3.8 Effect of SRS2 Scoial Cognition Score

### 3.8.1 Accuracy

#### 3.8.1.1 ASD

```
srs_sc_mod_asd <- glmer(correct ~ SRS_SocialCognition_scaled + (1|Case) + (1|pic), data = asd_dat, family = "binomial", control=glmerControl(optimizer="bobyqa"))
summary(srs_sc_mod_asd)
```

```
## Generalized linear mixed model fit by maximum likelihood (Laplace Approximation) ['glmerMod']
##  Family: binomial  ( logit )
## Formula: correct ~ SRS_SocialCognition_scaled + (1 | Case) + (1 | pic)
##    Data: asd_dat
## Control: glmerControl(optimizer = "bobyqa")
## 
##      AIC      BIC   logLik deviance df.resid 
##   5144.9   5171.0  -2568.4   5136.9     5100 
## 
## Scaled residuals: 
##     Min      1Q  Median      3Q     Max 
## -4.4101  0.2649  0.4078  0.5466  1.6529 
## 
## Random effects:
##  Groups Name        Variance Std.Dev.
##  Case   (Intercept) 0.3315   0.5757  
##  pic    (Intercept) 0.3117   0.5583  
## Number of obs: 5104, groups:  Case, 116; pic, 44
## 
## Fixed effects:
##                            Estimate Std. Error z value             Pr(>|z|)    
## (Intercept)                 1.40648    0.10690  13.157 < 0.0000000000000002 ***
## SRS_SocialCognition_scaled -0.21112    0.06394  -3.302              0.00096 ***
## ---
## Signif. codes:  0 '***' 0.001 '**' 0.01 '*' 0.05 '.' 0.1 ' ' 1
## 
## Correlation of Fixed Effects:
##             (Intr)
## SRS_SclCgn_ -0.018
```

```
Anova(srs_sc_mod_asd, type = 3)
```

```
## Analysis of Deviance Table (Type III Wald chisquare tests)
## 
## Response: correct
##                              Chisq Df            Pr(>Chisq)    
## (Intercept)                173.101  1 < 0.00000000000000022 ***
## SRS_SocialCognition_scaled  10.903  1             0.0009598 ***
## ---
## Signif. codes:  0 '***' 0.001 '**' 0.01 '*' 0.05 '.' 0.1 ' ' 1
```

```
check_model(srs_sc_mod_asd)
```

#### 3.8.1.2 NC

```
srs_sc_mod_nc <- glmer(correct ~ SRS_SocialCognition_scaled + (1|Case) + (1|pic), data = nc_dat, 
                        family = "binomial", control=glmerControl(optimizer="bobyqa"))
summary(srs_sc_mod_nc)
```

```
## Generalized linear mixed model fit by maximum likelihood (Laplace Approximation) ['glmerMod']
##  Family: binomial  ( logit )
## Formula: correct ~ SRS_SocialCognition_scaled + (1 | Case) + (1 | pic)
##    Data: nc_dat
## Control: glmerControl(optimizer = "bobyqa")
## 
##      AIC      BIC   logLik deviance df.resid 
##   3845.4   3871.4  -1918.7   3837.4     4879 
## 
## Scaled residuals: 
##     Min      1Q  Median      3Q     Max 
## -5.0038  0.2262  0.3253  0.4372  1.1703 
## 
## Random effects:
##  Groups Name        Variance Std.Dev.
##  Case   (Intercept) 0.1936   0.4400  
##  pic    (Intercept) 0.6045   0.7775  
## Number of obs: 4883, groups:  Case, 111; pic, 44
## 
## Fixed effects:
##                            Estimate Std. Error z value            Pr(>|z|)    
## (Intercept)                 2.01430    0.13428  15.001 <0.0000000000000002 ***
## SRS_SocialCognition_scaled -0.11391    0.05971  -1.908              0.0564 .  
## ---
## Signif. codes:  0 '***' 0.001 '**' 0.01 '*' 0.05 '.' 0.1 ' ' 1
## 
## Correlation of Fixed Effects:
##             (Intr)
## SRS_SclCgn_ -0.018
```

```
Anova(srs_sc_mod_nc, type = 3)
```

```
## Analysis of Deviance Table (Type III Wald chisquare tests)
## 
## Response: correct
##                               Chisq Df           Pr(>Chisq)    
## (Intercept)                225.0161  1 < 0.0000000000000002 ***
## SRS_SocialCognition_scaled   3.6388  1              0.05645 .  
## ---
## Signif. codes:  0 '***' 0.001 '**' 0.01 '*' 0.05 '.' 0.1 ' ' 1
```

```
check_model(srs_sc_mod_nc)
```

### 3.8.2 Response Time

#### 3.8.2.1 ASD

```
srs_sc_mod_rt_asd <- lmer(logRT ~ SRS_SocialCognition_scaled + (1|Case) + (1|pic), data = asd_rt_dat)
summary(srs_sc_mod_rt_asd)
```

```
## Linear mixed model fit by REML. t-tests use Satterthwaite's method ['lmerModLmerTest']
## Formula: logRT ~ SRS_SocialCognition_scaled + (1 | Case) + (1 | pic)
##    Data: asd_rt_dat
## 
## REML criterion at convergence: 4469.1
## 
## Scaled residuals: 
##     Min      1Q  Median      3Q     Max 
## -3.9548 -0.6499 -0.0481  0.5964  4.5001 
## 
## Random effects:
##  Groups   Name        Variance Std.Dev.
##  Case     (Intercept) 0.1387   0.3724  
##  pic      (Intercept) 0.0241   0.1553  
##  Residual             0.1590   0.3988  
## Number of obs: 3954, groups:  Case, 116; pic, 44
## 
## Fixed effects:
##                             Estimate Std. Error        df t value            Pr(>|t|)    
## (Intercept)                  7.99700    0.04226 148.11708 189.249 <0.0000000000000002 ***
## SRS_SocialCognition_scaled   0.03738    0.03538 114.55378   1.057               0.293    
## ---
## Signif. codes:  0 '***' 0.001 '**' 0.01 '*' 0.05 '.' 0.1 ' ' 1
## 
## Correlation of Fixed Effects:
##             (Intr)
## SRS_SclCgn_ 0.002
```

```
Anova(srs_sc_mod_rt_asd, type = 3)
```

```
## Analysis of Deviance Table (Type III Wald chisquare tests)
## 
## Response: logRT
##                                 Chisq Df          Pr(>Chisq)    
## (Intercept)                35815.1087  1 <0.0000000000000002 ***
## SRS_SocialCognition_scaled     1.1165  1              0.2907    
## ---
## Signif. codes:  0 '***' 0.001 '**' 0.01 '*' 0.05 '.' 0.1 ' ' 1
```

```
check_model(srs_sc_mod_rt_asd)
```

#### 3.8.2.2 NC

```
srs_sc_mod_rt_nc <- lmer(logRT ~ SRS_SocialCognition_scaled + (1|Case) + (1|pic), data = nc_rt_dat)
summary(srs_sc_mod_rt_nc)
```

```
## Linear mixed model fit by REML. t-tests use Satterthwaite's method ['lmerModLmerTest']
## Formula: logRT ~ SRS_SocialCognition_scaled + (1 | Case) + (1 | pic)
##    Data: nc_rt_dat
## 
## REML criterion at convergence: 3821
## 
## Scaled residuals: 
##     Min      1Q  Median      3Q     Max 
## -3.7619 -0.6766 -0.0654  0.6191  4.6886 
## 
## Random effects:
##  Groups   Name        Variance Std.Dev.
##  Case     (Intercept) 0.05928  0.2435  
##  pic      (Intercept) 0.01878  0.1371  
##  Residual             0.13182  0.3631  
## Number of obs: 4162, groups:  Case, 111; pic, 44
## 
## Fixed effects:
##                              Estimate Std. Error         df t value            Pr(>|t|)    
## (Intercept)                  7.826927   0.031517 126.369188 248.340 <0.0000000000000002 ***
## SRS_SocialCognition_scaled  -0.008916   0.023889 108.903603  -0.373                0.71    
## ---
## Signif. codes:  0 '***' 0.001 '**' 0.01 '*' 0.05 '.' 0.1 ' ' 1
## 
## Correlation of Fixed Effects:
##             (Intr)
## SRS_SclCgn_ 0.001
```

```
Anova(srs_sc_mod_rt_nc, type = 3)
```

```
## Analysis of Deviance Table (Type III Wald chisquare tests)
## 
## Response: logRT
##                                 Chisq Df          Pr(>Chisq)    
## (Intercept)                61672.8006  1 <0.0000000000000002 ***
## SRS_SocialCognition_scaled     0.1393  1               0.709    
## ---
## Signif. codes:  0 '***' 0.001 '**' 0.01 '*' 0.05 '.' 0.1 ' ' 1
```

```
check_model(srs_sc_mod_rt_nc)
```

## 3.9 Effect of SRS2 Scoial Communication Score

### 3.9.1 Accuracy

#### 3.9.1.1 ASD

```
srs_scom_mod_asd <- glmer(correct ~ SRS_SocialCommunication_scaled + (1|Case) + (1|pic), data = asd_dat, 
                          family = "binomial", control=glmerControl(optimizer="bobyqa"))
summary(srs_scom_mod_asd)
```

```
## Generalized linear mixed model fit by maximum likelihood (Laplace Approximation) ['glmerMod']
##  Family: binomial  ( logit )
## Formula: correct ~ SRS_SocialCommunication_scaled + (1 | Case) + (1 |      pic)
##    Data: asd_dat
## Control: glmerControl(optimizer = "bobyqa")
## 
##      AIC      BIC   logLik deviance df.resid 
##   5150.5   5176.7  -2571.3   5142.5     5100 
## 
## Scaled residuals: 
##     Min      1Q  Median      3Q     Max 
## -4.3428  0.2639  0.4073  0.5461  1.6793 
## 
## Random effects:
##  Groups Name        Variance Std.Dev.
##  Case   (Intercept) 0.3577   0.5981  
##  pic    (Intercept) 0.3118   0.5584  
## Number of obs: 5104, groups:  Case, 116; pic, 44
## 
## Fixed effects:
##                                Estimate Std. Error z value            Pr(>|z|)    
## (Intercept)                     1.40719    0.10796  13.034 <0.0000000000000002 ***
## SRS_SocialCommunication_scaled -0.14469    0.06593  -2.194              0.0282 *  
## ---
## Signif. codes:  0 '***' 0.001 '**' 0.01 '*' 0.05 '.' 0.1 ' ' 1
## 
## Correlation of Fixed Effects:
##             (Intr)
## SRS_SclCmm_ -0.012
```

```
Anova(srs_scom_mod_asd, type = 3)
```

```
## Analysis of Deviance Table (Type III Wald chisquare tests)
## 
## Response: correct
##                                   Chisq Df          Pr(>Chisq)    
## (Intercept)                    169.8841  1 <0.0000000000000002 ***
## SRS_SocialCommunication_scaled   4.8157  1              0.0282 *  
## ---
## Signif. codes:  0 '***' 0.001 '**' 0.01 '*' 0.05 '.' 0.1 ' ' 1
```

```
check_model(srs_scom_mod_asd)
```

#### 3.9.1.2 NC

```
srs_scom_mod_nc <- glmer(correct ~ SRS_SocialCommunication_scaled + (1|Case) + (1|pic), data = nc_dat, 
                        family = "binomial", control=glmerControl(optimizer="bobyqa"))
summary(srs_scom_mod_nc)
```

```
## Generalized linear mixed model fit by maximum likelihood (Laplace Approximation) ['glmerMod']
##  Family: binomial  ( logit )
## Formula: correct ~ SRS_SocialCommunication_scaled + (1 | Case) + (1 |      pic)
##    Data: nc_dat
## Control: glmerControl(optimizer = "bobyqa")
## 
##      AIC      BIC   logLik deviance df.resid 
##     3842     3868    -1917     3834     4879 
## 
## Scaled residuals: 
##     Min      1Q  Median      3Q     Max 
## -5.1933  0.2253  0.3259  0.4352  1.1577 
## 
## Random effects:
##  Groups Name        Variance Std.Dev.
##  Case   (Intercept) 0.1807   0.4250  
##  pic    (Intercept) 0.6045   0.7775  
## Number of obs: 4883, groups:  Case, 111; pic, 44
## 
## Fixed effects:
##                                Estimate Std. Error z value             Pr(>|z|)    
## (Intercept)                     2.01403    0.13385  15.046 < 0.0000000000000002 ***
## SRS_SocialCommunication_scaled -0.15812    0.05837  -2.709              0.00675 ** 
## ---
## Signif. codes:  0 '***' 0.001 '**' 0.01 '*' 0.05 '.' 0.1 ' ' 1
## 
## Correlation of Fixed Effects:
##             (Intr)
## SRS_SclCmm_ -0.025
```

```
Anova(srs_scom_mod_nc, type = 3)
```

```
## Analysis of Deviance Table (Type III Wald chisquare tests)
## 
## Response: correct
##                                   Chisq Df            Pr(>Chisq)    
## (Intercept)                    226.3962  1 < 0.00000000000000022 ***
## SRS_SocialCommunication_scaled   7.3379  1              0.006751 ** 
## ---
## Signif. codes:  0 '***' 0.001 '**' 0.01 '*' 0.05 '.' 0.1 ' ' 1
```

```
check_model(srs_scom_mod_nc)
```

### 3.9.2 Response Time

#### 3.9.2.1 ASD

```
srs_scom_mod_rt_asd <- lmer(logRT ~ SRS_SocialCommunication_scaled + (1|Case) + (1|pic), data = asd_rt_dat)
summary(srs_scom_mod_rt_asd)
```

```
## Linear mixed model fit by REML. t-tests use Satterthwaite's method ['lmerModLmerTest']
## Formula: logRT ~ SRS_SocialCommunication_scaled + (1 | Case) + (1 | pic)
##    Data: asd_rt_dat
## 
## REML criterion at convergence: 4469.5
## 
## Scaled residuals: 
##     Min      1Q  Median      3Q     Max 
## -3.9518 -0.6490 -0.0482  0.5975  4.4969 
## 
## Random effects:
##  Groups   Name        Variance Std.Dev.
##  Case     (Intercept) 0.13925  0.3732  
##  pic      (Intercept) 0.02411  0.1553  
##  Residual             0.15902  0.3988  
## Number of obs: 3954, groups:  Case, 116; pic, 44
## 
## Fixed effects:
##                                 Estimate Std. Error        df t value            Pr(>|t|)    
## (Intercept)                      7.99697    0.04231 148.17232 189.000 <0.0000000000000002 ***
## SRS_SocialCommunication_scaled   0.02910    0.03542 114.23220   0.822               0.413    
## ---
## Signif. codes:  0 '***' 0.001 '**' 0.01 '*' 0.05 '.' 0.1 ' ' 1
## 
## Correlation of Fixed Effects:
##             (Intr)
## SRS_SclCmm_ 0.001
```

```
Anova(srs_scom_mod_rt_asd, type = 3)
```

```
## Analysis of Deviance Table (Type III Wald chisquare tests)
## 
## Response: logRT
##                                     Chisq Df          Pr(>Chisq)    
## (Intercept)                    35721.1540  1 <0.0000000000000002 ***
## SRS_SocialCommunication_scaled     0.6749  1              0.4113    
## ---
## Signif. codes:  0 '***' 0.001 '**' 0.01 '*' 0.05 '.' 0.1 ' ' 1
```

```
check_model(srs_scom_mod_rt_asd)
```

#### 3.9.2.2 NC

```
srs_scom_mod_rt_nc <- lmer(logRT ~ SRS_SocialCommunication_scaled + (1|Case) + (1|pic), data = nc_rt_dat)
summary(srs_scom_mod_rt_nc)
```

```
## Linear mixed model fit by REML. t-tests use Satterthwaite's method ['lmerModLmerTest']
## Formula: logRT ~ SRS_SocialCommunication_scaled + (1 | Case) + (1 | pic)
##    Data: nc_rt_dat
## 
## REML criterion at convergence: 3821
## 
## Scaled residuals: 
##     Min      1Q  Median      3Q     Max 
## -3.7612 -0.6764 -0.0655  0.6172  4.6849 
## 
## Random effects:
##  Groups   Name        Variance Std.Dev.
##  Case     (Intercept) 0.05931  0.2435  
##  pic      (Intercept) 0.01878  0.1371  
##  Residual             0.13182  0.3631  
## Number of obs: 4162, groups:  Case, 111; pic, 44
## 
## Fixed effects:
##                                  Estimate Std. Error         df t value            Pr(>|t|)    
## (Intercept)                      7.826945   0.031521 126.394664   248.3 <0.0000000000000002 ***
## SRS_SocialCommunication_scaled   0.007165   0.023903 109.070245     0.3               0.765    
## ---
## Signif. codes:  0 '***' 0.001 '**' 0.01 '*' 0.05 '.' 0.1 ' ' 1
## 
## Correlation of Fixed Effects:
##             (Intr)
## SRS_SclCmm_ 0.001
```

```
Anova(srs_scom_mod_rt_nc, type = 3)
```

```
## Analysis of Deviance Table (Type III Wald chisquare tests)
## 
## Response: logRT
##                                     Chisq Df          Pr(>Chisq)    
## (Intercept)                    61657.0233  1 <0.0000000000000002 ***
## SRS_SocialCommunication_scaled     0.0899  1              0.7644    
## ---
## Signif. codes:  0 '***' 0.001 '**' 0.01 '*' 0.05 '.' 0.1 ' ' 1
```

```
check_model(srs_scom_mod_rt_nc)
```

## 3.10 Effect of SRS2 Scoial Motivation Score

### 3.10.1 Accuracy

#### 3.10.1.1 ASD

```
srs_sm_mod_asd <- glmer(correct ~ SRS_SocialMotivation_scaled + (1|Case) + (1|pic), data = asd_dat, 
                        family = "binomial", control=glmerControl(optimizer="bobyqa"))
summary(srs_sm_mod_asd)
```

```
## Generalized linear mixed model fit by maximum likelihood (Laplace Approximation) ['glmerMod']
##  Family: binomial  ( logit )
## Formula: correct ~ SRS_SocialMotivation_scaled + (1 | Case) + (1 | pic)
##    Data: asd_dat
## Control: glmerControl(optimizer = "bobyqa")
## 
##      AIC      BIC   logLik deviance df.resid 
##   5152.9   5179.1  -2572.5   5144.9     5100 
## 
## Scaled residuals: 
##     Min      1Q  Median      3Q     Max 
## -4.4270  0.2654  0.4091  0.5455  1.6782 
## 
## Random effects:
##  Groups Name        Variance Std.Dev.
##  Case   (Intercept) 0.3679   0.6065  
##  pic    (Intercept) 0.3118   0.5584  
## Number of obs: 5104, groups:  Case, 116; pic, 44
## 
## Fixed effects:
##                             Estimate Std. Error z value            Pr(>|z|)    
## (Intercept)                  1.40712    0.10837   12.98 <0.0000000000000002 ***
## SRS_SocialMotivation_scaled -0.10187    0.06702   -1.52               0.129    
## ---
## Signif. codes:  0 '***' 0.001 '**' 0.01 '*' 0.05 '.' 0.1 ' ' 1
## 
## Correlation of Fixed Effects:
##             (Intr)
## SRS_SclMtv_ -0.006
```

```
Anova(srs_sm_mod_asd, type = 3)
```

```
## Analysis of Deviance Table (Type III Wald chisquare tests)
## 
## Response: correct
##                                Chisq Df          Pr(>Chisq)    
## (Intercept)                 168.5983  1 <0.0000000000000002 ***
## SRS_SocialMotivation_scaled   2.3104  1              0.1285    
## ---
## Signif. codes:  0 '***' 0.001 '**' 0.01 '*' 0.05 '.' 0.1 ' ' 1
```

```
check_model(srs_sm_mod_asd)
```

#### 3.10.1.2 NC

```
srs_sm_mod_nc <- glmer(correct ~ SRS_SocialMotivation_scaled + (1|Case) + (1|pic), data = nc_dat, 
                       family = "binomial", control=glmerControl(optimizer="bobyqa"))
summary(srs_sm_mod_nc)
```

```
## Generalized linear mixed model fit by maximum likelihood (Laplace Approximation) ['glmerMod']
##  Family: binomial  ( logit )
## Formula: correct ~ SRS_SocialMotivation_scaled + (1 | Case) + (1 | pic)
##    Data: nc_dat
## Control: glmerControl(optimizer = "bobyqa")
## 
##      AIC      BIC   logLik deviance df.resid 
##   3848.9   3874.8  -1920.4   3840.9     4879 
## 
## Scaled residuals: 
##     Min      1Q  Median      3Q     Max 
## -5.0495  0.2267  0.3259  0.4361  1.1720 
## 
## Random effects:
##  Groups Name        Variance Std.Dev.
##  Case   (Intercept) 0.2057   0.4536  
##  pic    (Intercept) 0.6045   0.7775  
## Number of obs: 4883, groups:  Case, 111; pic, 44
## 
## Fixed effects:
##                             Estimate Std. Error z value            Pr(>|z|)    
## (Intercept)                  2.01420    0.13468  14.955 <0.0000000000000002 ***
## SRS_SocialMotivation_scaled -0.01934    0.06059  -0.319                0.75    
## ---
## Signif. codes:  0 '***' 0.001 '**' 0.01 '*' 0.05 '.' 0.1 ' ' 1
## 
## Correlation of Fixed Effects:
##             (Intr)
## SRS_SclMtv_ -0.002
```

```
Anova(srs_sm_mod_nc, type = 3)
```

```
## Analysis of Deviance Table (Type III Wald chisquare tests)
## 
## Response: correct
##                                Chisq Df          Pr(>Chisq)    
## (Intercept)                 223.6655  1 <0.0000000000000002 ***
## SRS_SocialMotivation_scaled   0.1019  1              0.7496    
## ---
## Signif. codes:  0 '***' 0.001 '**' 0.01 '*' 0.05 '.' 0.1 ' ' 1
```

```
check_model(srs_sm_mod_nc)
```

### 3.10.2 Response Time

#### 3.10.2.1 ASD

```
srs_sm_mod_rt_asd <- lmer(logRT ~ SRS_SocialMotivation_scaled + (1|Case) + (1|pic), data = asd_rt_dat)
summary(srs_sm_mod_rt_asd)
```

```
## Linear mixed model fit by REML. t-tests use Satterthwaite's method ['lmerModLmerTest']
## Formula: logRT ~ SRS_SocialMotivation_scaled + (1 | Case) + (1 | pic)
##    Data: asd_rt_dat
## 
## REML criterion at convergence: 4470
## 
## Scaled residuals: 
##     Min      1Q  Median      3Q     Max 
## -3.9477 -0.6489 -0.0482  0.5970  4.4990 
## 
## Random effects:
##  Groups   Name        Variance Std.Dev.
##  Case     (Intercept) 0.13993  0.3741  
##  pic      (Intercept) 0.02411  0.1553  
##  Residual             0.15902  0.3988  
## Number of obs: 3954, groups:  Case, 116; pic, 44
## 
## Fixed effects:
##                              Estimate Std. Error        df t value            Pr(>|t|)    
## (Intercept)                   7.99694    0.04238 148.29746 188.690 <0.0000000000000002 ***
## SRS_SocialMotivation_scaled   0.01393    0.03549 114.08804   0.392               0.696    
## ---
## Signif. codes:  0 '***' 0.001 '**' 0.01 '*' 0.05 '.' 0.1 ' ' 1
## 
## Correlation of Fixed Effects:
##             (Intr)
## SRS_SclMtv_ 0.001
```

```
Anova(srs_sm_mod_rt_asd, type = 3)
```

```
## Analysis of Deviance Table (Type III Wald chisquare tests)
## 
## Response: logRT
##                                 Chisq Df          Pr(>Chisq)    
## (Intercept)                 35604.097  1 <0.0000000000000002 ***
## SRS_SocialMotivation_scaled     0.154  1              0.6948    
## ---
## Signif. codes:  0 '***' 0.001 '**' 0.01 '*' 0.05 '.' 0.1 ' ' 1
```

```
check_model(srs_sm_mod_rt_asd)
```

#### 3.10.2.2 NC

```
srs_sm_mod_rt_nc <- lmer(logRT ~ SRS_SocialMotivation_scaled + (1|Case) + (1|pic), data = nc_rt_dat)
summary(srs_sm_mod_rt_nc)
```

```
## Linear mixed model fit by REML. t-tests use Satterthwaite's method ['lmerModLmerTest']
## Formula: logRT ~ SRS_SocialMotivation_scaled + (1 | Case) + (1 | pic)
##    Data: nc_rt_dat
## 
## REML criterion at convergence: 3820.6
## 
## Scaled residuals: 
##     Min      1Q  Median      3Q     Max 
## -3.7628 -0.6759 -0.0659  0.6179  4.6899 
## 
## Random effects:
##  Groups   Name        Variance Std.Dev.
##  Case     (Intercept) 0.05905  0.2430  
##  pic      (Intercept) 0.01878  0.1371  
##  Residual             0.13182  0.3631  
## Number of obs: 4162, groups:  Case, 111; pic, 44
## 
## Fixed effects:
##                              Estimate Std. Error        df t value            Pr(>|t|)    
## (Intercept)                   7.82693    0.03148 126.19217 248.598 <0.0000000000000002 ***
## SRS_SocialMotivation_scaled  -0.01749    0.02385 109.05176  -0.733               0.465    
## ---
## Signif. codes:  0 '***' 0.001 '**' 0.01 '*' 0.05 '.' 0.1 ' ' 1
## 
## Correlation of Fixed Effects:
##             (Intr)
## SRS_SclMtv_ 0.000
```

```
Anova(srs_sm_mod_rt_nc, type = 3)
```

```
## Analysis of Deviance Table (Type III Wald chisquare tests)
## 
## Response: logRT
##                                  Chisq Df          Pr(>Chisq)    
## (Intercept)                 61800.7848  1 <0.0000000000000002 ***
## SRS_SocialMotivation_scaled     0.5373  1              0.4635    
## ---
## Signif. codes:  0 '***' 0.001 '**' 0.01 '*' 0.05 '.' 0.1 ' ' 1
```

```
check_model(srs_sm_mod_rt_nc)
```

## 3.11 Effect of SRS2 Scoial Restrictive & Repetitive Behaviors

### 3.11.1 Accuracy

#### 3.11.1.1 ASD

```
srs_am_mod_asd <- glmer(correct ~ SRS_AutisticMannerism_scaled + (1|Case) + (1|pic), data = asd_dat, 
                        family = "binomial", control=glmerControl(optimizer="bobyqa"))
summary(srs_am_mod_asd)
```

```
## Generalized linear mixed model fit by maximum likelihood (Laplace Approximation) ['glmerMod']
##  Family: binomial  ( logit )
## Formula: correct ~ SRS_AutisticMannerism_scaled + (1 | Case) + (1 | pic)
##    Data: asd_dat
## Control: glmerControl(optimizer = "bobyqa")
## 
##      AIC      BIC   logLik deviance df.resid 
##   5151.9   5178.1  -2572.0   5143.9     5100 
## 
## Scaled residuals: 
##     Min      1Q  Median      3Q     Max 
## -4.4510  0.2624  0.4084  0.5466  1.6662 
## 
## Random effects:
##  Groups Name        Variance Std.Dev.
##  Case   (Intercept) 0.3649   0.6041  
##  pic    (Intercept) 0.3118   0.5584  
## Number of obs: 5104, groups:  Case, 116; pic, 44
## 
## Fixed effects:
##                              Estimate Std. Error z value            Pr(>|z|)    
## (Intercept)                   1.40748    0.10826  13.001 <0.0000000000000002 ***
## SRS_AutisticMannerism_scaled -0.12193    0.06682  -1.825              0.0681 .  
## ---
## Signif. codes:  0 '***' 0.001 '**' 0.01 '*' 0.05 '.' 0.1 ' ' 1
## 
## Correlation of Fixed Effects:
##             (Intr)
## SRS_AtstcM_ -0.010
```

```
Anova(srs_am_mod_asd, type = 3)
```

```
## Analysis of Deviance Table (Type III Wald chisquare tests)
## 
## Response: correct
##                                 Chisq Df           Pr(>Chisq)    
## (Intercept)                  169.0358  1 < 0.0000000000000002 ***
## SRS_AutisticMannerism_scaled   3.3291  1              0.06806 .  
## ---
## Signif. codes:  0 '***' 0.001 '**' 0.01 '*' 0.05 '.' 0.1 ' ' 1
```

```
check_model(srs_am_mod_asd)
```

#### 3.11.1.2 NC

```
srs_am_mod_nc <- glmer(correct ~ SRS_AutisticMannerism_scaled + (1|Case) + (1|pic), data = nc_dat, 
                       family = "binomial", control=glmerControl(optimizer="bobyqa"))
summary(srs_am_mod_nc)
```

```
## Generalized linear mixed model fit by maximum likelihood (Laplace Approximation) ['glmerMod']
##  Family: binomial  ( logit )
## Formula: correct ~ SRS_AutisticMannerism_scaled + (1 | Case) + (1 | pic)
##    Data: nc_dat
## Control: glmerControl(optimizer = "bobyqa")
## 
##      AIC      BIC   logLik deviance df.resid 
##   3846.3   3872.3  -1919.2   3838.3     4879 
## 
## Scaled residuals: 
##     Min      1Q  Median      3Q     Max 
## -5.1119  0.2267  0.3251  0.4375  1.1995 
## 
## Random effects:
##  Groups Name        Variance Std.Dev.
##  Case   (Intercept) 0.1971   0.4439  
##  pic    (Intercept) 0.6046   0.7776  
## Number of obs: 4883, groups:  Case, 111; pic, 44
## 
## Fixed effects:
##                              Estimate Std. Error z value            Pr(>|z|)    
## (Intercept)                   2.01432    0.13441  14.987 <0.0000000000000002 ***
## SRS_AutisticMannerism_scaled -0.09756    0.05904  -1.652              0.0985 .  
## ---
## Signif. codes:  0 '***' 0.001 '**' 0.01 '*' 0.05 '.' 0.1 ' ' 1
## 
## Correlation of Fixed Effects:
##             (Intr)
## SRS_AtstcM_ -0.016
```

```
Anova(srs_am_mod_nc, type = 3)
```

```
## Analysis of Deviance Table (Type III Wald chisquare tests)
## 
## Response: correct
##                                 Chisq Df           Pr(>Chisq)    
## (Intercept)                  224.5978  1 < 0.0000000000000002 ***
## SRS_AutisticMannerism_scaled   2.7303  1              0.09846 .  
## ---
## Signif. codes:  0 '***' 0.001 '**' 0.01 '*' 0.05 '.' 0.1 ' ' 1
```

```
check_model(srs_am_mod_nc)
```

### 3.11.2 Response Time

#### 3.11.2.1 ASD

```
srs_am_mod_rt_asd <- lmer(logRT ~ SRS_AutisticMannerism_scaled + (1|Case) + (1|pic), data = asd_rt_dat)
summary(srs_am_mod_rt_asd)
```

```
## Linear mixed model fit by REML. t-tests use Satterthwaite's method ['lmerModLmerTest']
## Formula: logRT ~ SRS_AutisticMannerism_scaled + (1 | Case) + (1 | pic)
##    Data: asd_rt_dat
## 
## REML criterion at convergence: 4469.6
## 
## Scaled residuals: 
##     Min      1Q  Median      3Q     Max 
## -3.9484 -0.6506 -0.0470  0.5974  4.4980 
## 
## Random effects:
##  Groups   Name        Variance Std.Dev.
##  Case     (Intercept) 0.1394   0.3734  
##  pic      (Intercept) 0.0241   0.1552  
##  Residual             0.1590   0.3988  
## Number of obs: 3954, groups:  Case, 116; pic, 44
## 
## Fixed effects:
##                               Estimate Std. Error        df t value            Pr(>|t|)    
## (Intercept)                    7.99696    0.04233 148.21025 188.928 <0.0000000000000002 ***
## SRS_AutisticMannerism_scaled   0.02641    0.03542 114.00883   0.746               0.457    
## ---
## Signif. codes:  0 '***' 0.001 '**' 0.01 '*' 0.05 '.' 0.1 ' ' 1
## 
## Correlation of Fixed Effects:
##             (Intr)
## SRS_AtstcM_ 0.001
```

```
Anova(srs_am_mod_rt_asd, type = 3)
```

```
## Analysis of Deviance Table (Type III Wald chisquare tests)
## 
## Response: logRT
##                                   Chisq Df          Pr(>Chisq)    
## (Intercept)                  35693.9681  1 <0.0000000000000002 ***
## SRS_AutisticMannerism_scaled     0.5561  1              0.4559    
## ---
## Signif. codes:  0 '***' 0.001 '**' 0.01 '*' 0.05 '.' 0.1 ' ' 1
```

```
check_model(srs_am_mod_rt_asd)
```

#### 3.11.2.2 NC

```
srs_am_mod_rt_nc <- lmer(logRT ~ SRS_AutisticMannerism_scaled + (1|Case) + (1|pic), data = nc_rt_dat)
summary(srs_am_mod_rt_nc)
```

```
## Linear mixed model fit by REML. t-tests use Satterthwaite's method ['lmerModLmerTest']
## Formula: logRT ~ SRS_AutisticMannerism_scaled + (1 | Case) + (1 | pic)
##    Data: nc_rt_dat
## 
## REML criterion at convergence: 3820.8
## 
## Scaled residuals: 
##     Min      1Q  Median      3Q     Max 
## -3.7612 -0.6761 -0.0658  0.6183  4.6842 
## 
## Random effects:
##  Groups   Name        Variance Std.Dev.
##  Case     (Intercept) 0.05919  0.2433  
##  pic      (Intercept) 0.01878  0.1370  
##  Residual             0.13182  0.3631  
## Number of obs: 4162, groups:  Case, 111; pic, 44
## 
## Fixed effects:
##                               Estimate Std. Error        df t value            Pr(>|t|)    
## (Intercept)                    7.82695    0.03150 126.30491 248.449 <0.0000000000000002 ***
## SRS_AutisticMannerism_scaled   0.01313    0.02390 109.39595   0.549               0.584    
## ---
## Signif. codes:  0 '***' 0.001 '**' 0.01 '*' 0.05 '.' 0.1 ' ' 1
## 
## Correlation of Fixed Effects:
##             (Intr)
## SRS_AtstcM_ 0.001
```

```
Anova(srs_am_mod_rt_nc, type = 3)
```

```
## Analysis of Deviance Table (Type III Wald chisquare tests)
## 
## Response: logRT
##                                   Chisq Df          Pr(>Chisq)    
## (Intercept)                  61726.6834  1 <0.0000000000000002 ***
## SRS_AutisticMannerism_scaled     0.3018  1              0.5828    
## ---
## Signif. codes:  0 '***' 0.001 '**' 0.01 '*' 0.05 '.' 0.1 ' ' 1
```

```
check_model(srs_am_mod_rt_nc)
```

## 3.12 Interaction Effects with Social-Cognitive Traits in ASD (on Accuracy)

### 3.12.1 Trial-Number

```
asd_trial_srs_mod <- glmer(correct ~ Trial_scaled * SRS_SocialCognition_scaled + (1|Case) + (1|pic), data = asd_dat, 
                           family = "binomial", control=glmerControl(optimizer="bobyqa"))
summary(asd_trial_srs_mod)
```

```
## Generalized linear mixed model fit by maximum likelihood (Laplace Approximation) ['glmerMod']
##  Family: binomial  ( logit )
## Formula: correct ~ Trial_scaled * SRS_SocialCognition_scaled + (1 | Case) +      (1 | pic)
##    Data: asd_dat
## Control: glmerControl(optimizer = "bobyqa")
## 
##      AIC      BIC   logLik deviance df.resid 
##   5138.6   5177.8  -2563.3   5126.6     5098 
## 
## Scaled residuals: 
##     Min      1Q  Median      3Q     Max 
## -4.5561  0.2592  0.4071  0.5440  1.6448 
## 
## Random effects:
##  Groups Name        Variance Std.Dev.
##  Case   (Intercept) 0.3337   0.5777  
##  pic    (Intercept) 0.3147   0.5610  
## Number of obs: 5104, groups:  Case, 116; pic, 44
## 
## Fixed effects:
##                                         Estimate Std. Error z value             Pr(>|z|)    
## (Intercept)                              1.41011    0.10735  13.136 < 0.0000000000000002 ***
## Trial_scaled                             0.10956    0.03596   3.046              0.00232 ** 
## SRS_SocialCognition_scaled              -0.21063    0.06413  -3.285              0.00102 ** 
## Trial_scaled:SRS_SocialCognition_scaled  0.02768    0.03465   0.799              0.42447    
## ---
## Signif. codes:  0 '***' 0.001 '**' 0.01 '*' 0.05 '.' 0.1 ' ' 1
## 
## Correlation of Fixed Effects:
##             (Intr) Trl_sc SRS_SC
## Trial_scald  0.020              
## SRS_SclCgn_ -0.018  0.000       
## Tr_:SRS_SC_  0.001 -0.081  0.019
```

```
Anova(asd_trial_srs_mod, type = 3)
```

```
## Analysis of Deviance Table (Type III Wald chisquare tests)
## 
## Response: correct
##                                            Chisq Df            Pr(>Chisq)    
## (Intercept)                             172.5585  1 < 0.00000000000000022 ***
## Trial_scaled                              9.2806  1              0.002316 ** 
## SRS_SocialCognition_scaled               10.7881  1              0.001022 ** 
## Trial_scaled:SRS_SocialCognition_scaled   0.6379  1              0.424470    
## ---
## Signif. codes:  0 '***' 0.001 '**' 0.01 '*' 0.05 '.' 0.1 ' ' 1
```

```
check_model(asd_trial_srs_mod)
```

### 3.12.2 Contextual Ambiguity (Target-Distractor Delta)

```
asd_delta_srs_mod <- glmer(correct ~ delta * SRS_SocialCognition_scaled + (1|Case) + (1|pic), data = asd_dat, 
                           family = "binomial", control=glmerControl(optimizer="bobyqa"))
summary(asd_delta_srs_mod)
```

```
## Generalized linear mixed model fit by maximum likelihood (Laplace Approximation) ['glmerMod']
##  Family: binomial  ( logit )
## Formula: correct ~ delta * SRS_SocialCognition_scaled + (1 | Case) + (1 |      pic)
##    Data: asd_dat
## Control: glmerControl(optimizer = "bobyqa")
## 
##      AIC      BIC   logLik deviance df.resid 
##   5144.4   5183.6  -2566.2   5132.4     5098 
## 
## Scaled residuals: 
##     Min      1Q  Median      3Q     Max 
## -4.3762  0.2606  0.4066  0.5467  1.6636 
## 
## Random effects:
##  Groups Name        Variance Std.Dev.
##  Case   (Intercept) 0.3316   0.5758  
##  pic    (Intercept) 0.3098   0.5566  
## Number of obs: 5104, groups:  Case, 116; pic, 44
## 
## Fixed effects:
##                                  Estimate Std. Error z value            Pr(>|z|)    
## (Intercept)                       1.34136    0.14130   9.493 <0.0000000000000002 ***
## delta                             0.15568    0.21700   0.717              0.4731    
## SRS_SocialCognition_scaled       -0.14245    0.07223  -1.972              0.0486 *  
## delta:SRS_SocialCognition_scaled -0.16510    0.08097  -2.039              0.0414 *  
## ---
## Signif. codes:  0 '***' 0.001 '**' 0.01 '*' 0.05 '.' 0.1 ' ' 1
## 
## Correlation of Fixed Effects:
##             (Intr) delta  SRS_SC
## delta       -0.655              
## SRS_SclCgn_ -0.014  0.010       
## dlt:SRS_SC_  0.013 -0.035 -0.464
```

```
Anova(asd_delta_srs_mod, type = 3)
```

```
## Analysis of Deviance Table (Type III Wald chisquare tests)
## 
## Response: correct
##                                    Chisq Df           Pr(>Chisq)    
## (Intercept)                      90.1217  1 < 0.0000000000000002 ***
## delta                             0.5147  1              0.47311    
## SRS_SocialCognition_scaled        3.8898  1              0.04858 *  
## delta:SRS_SocialCognition_scaled  4.1577  1              0.04145 *  
## ---
## Signif. codes:  0 '***' 0.001 '**' 0.01 '*' 0.05 '.' 0.1 ' ' 1
```

```
check_model(asd_delta_srs_mod)
```

# 4 References

**The versions of all packaged used in this Rmd file were:**

```
# Get the list of all loaded packages
loaded_packages <- sessionInfo()$otherPkgs

# Define a function to extract and format the citation for a single package
generate_citation <- function(pkg_name, pkg_info) {
  # Retrieve the citation information
  citation_info <- citation(pkg_name)
  
  # Handle multiple citations: select the one with a URL (assuming it's the package citation)
  if (length(citation_info) > 1) {
    # Identify citations that contain a URL
    pkg_citations_with_url <- citation_info[sapply(citation_info, function(x) !is.null(x$url))]
    
    if (length(pkg_citations_with_url) > 0) {
      # Select the first citation with a URL
      pkg_citation <- pkg_citations_with_url[[1]]
    } else {
      # If no citation has a URL, default to the first citation
      pkg_citation <- citation_info[[1]]
    }
  } else {
    # Only one citation exists
    pkg_citation <- citation_info[[1]]
  }
  
  # Extract package name
  package_name <- pkg_name
  
  # Extract version information
  version <- pkg_info$Version
  if (is.null(version) || version == "") {
    version <- as.character(packageVersion(pkg_name))
  }
  
  # Extract and format authors
  if (!is.null(pkg_citation$author)) {
    # Use 'format' to convert 'person' objects to readable strings
    authors <- paste(format(pkg_citation$author), collapse = ", ")
  } else {
    authors <- "Unknown Author"
  }
  
  # Extract title
  title <- pkg_citation$title
  if (is.null(title) || title == "") {
    title <- "No title available"
  }
  
  # Extract year
  year <- pkg_citation$year
  if (is.null(year) || year == "") {
    year <- "Unknown Year"
  }
  
  # Extract URL, provide a default if missing
  if (!is.null(pkg_citation$url) && nzchar(pkg_citation$url)) {
    url <- pkg_citation$url
  } else {
    # Construct a default CRAN URL
    url <- paste0("https://CRAN.R-project.org/package=", pkg_name)
  }
  
  # Format the citation as desired
  citation_text <- paste0(
    package_name, ", version ", version, ", ",
    authors, " (", year, "). ",
    title, ". R package version ", version, ", <", url, ">."
  )
  
  return(citation_text)
}

# Generate the citation for R itself
r_citation <- citation()

# Assuming the first citation is the primary one for R itself
r_citation_text <- paste0(
  "R, version ", getRversion(), ", ",
  paste(format(r_citation$author), collapse = ", "), " (", r_citation$year, "). ",
  r_citation$title, ". ", r_citation$note, ", <", r_citation$url, ">."
)

# Apply the function to all loaded packages
# Initialize an empty vector to store citations
all_citations <- character(0)

# Add the R citation at the top
all_citations <- c(all_citations, r_citation_text)

# Loop through each loaded package and generate its citation
for (pkg_name in names(loaded_packages)) {
  pkg_info <- loaded_packages[[pkg_name]]
  citation_text <- generate_citation(pkg_name, pkg_info)
  all_citations <- c(all_citations, citation_text)
}

# Print the citations
cat(paste(all_citations, collapse = "\n\n"))
```

```
## R, version 4.2.2, R Core Team (2022). R: A Language and Environment for Statistical Computing. , <https://www.R-project.org/>.
## 
## kableExtra, version 1.4.0, Hao Zhu <haozhu233@gmail.com> [aut, cre] (<https://orcid.org/0000-0002-3386-6076>) (2024). kableExtra: Construct Complex Table with 'kable' and Pipe Syntax. R package version 1.4.0, <https://CRAN.R-project.org/package=kableExtra>.
## 
## performance, version 0.14.0, Daniel Lüdecke, Mattan S. Ben-Shachar, Indrajeet Patil, Philip Waggoner, Dominique Makowski (2021). {performance}: An {R} Package for Assessment, Comparison and Testing of Statistical Models. R package version 0.14.0, <https://CRAN.R-project.org/package=performance>.
## 
## effects, version 4.2-2, John Fox, Sanford Weisberg (2019). An R Companion to Applied Regression. R package version 4.2-2, <https://socialsciences.mcmaster.ca/jfox/Books/Companion/index.html>.
## 
## car, version 3.1-1, John Fox, Sanford Weisberg (2019). An {R} Companion to Applied Regression. R package version 3.1-1, <https://socialsciences.mcmaster.ca/jfox/Books/Companion/>.
## 
## carData, version 3.0-5, John Fox <jfox@mcmaster.ca> [aut, cre], Sanford Weisberg <sandy@umn.edu> [aut], Brad Price <brad.price@mail.wvu.edu> [aut] (2022). carData: Companion to Applied Regression Data Sets. R package version 3.0-5, <https://CRAN.R-project.org/package=carData>.
## 
## lmerTest, version 3.1-3, Alexandra Kuznetsova <alku@dtu.dk>, Per B. Brockhoff <perbb@dtu.dk>, Rune H. B. Christensen <Rune@ChristensenStatistics.dk> (2017). {lmerTest} Package: Tests in Linear Mixed Effects Models. R package version 3.1-3, <https://CRAN.R-project.org/package=lmerTest>.
## 
## lme4, version 1.1-31, Douglas Bates, Martin M{\"a}chler, Ben Bolker, Steve Walker (2015). Fitting Linear Mixed-Effects Models Using {lme4}. R package version 1.1-31, <https://CRAN.R-project.org/package=lme4>.
## 
## Matrix, version 1.5-1, Douglas Bates [aut], Martin Maechler <mmaechler+Matrix@gmail.com> [aut, cre] (<https://orcid.org/0000-0002-8685-9910>), Mikael Jagan [aut] (<https://orcid.org/0000-0002-3542-2938>) (2022). Matrix: Sparse and Dense Matrix Classes and Methods. R package version 1.5-1, <https://CRAN.R-project.org/package=Matrix>.
## 
## forcats, version 1.0.0, Hadley Wickham <hadley@rstudio.com> [aut, cre] (2023). forcats: Tools for Working with Categorical Variables (Factors). R package version 1.0.0, <https://CRAN.R-project.org/package=forcats>.
## 
## stringr, version 1.5.0, Hadley Wickham <hadley@rstudio.com> [aut, cre, cph] (2022). stringr: Simple, Consistent Wrappers for Common String Operations. R package version 1.5.0, <https://CRAN.R-project.org/package=stringr>.
## 
## dplyr, version 1.1.3, Hadley Wickham <hadley@posit.co> [aut, cre] (<https://orcid.org/0000-0003-4757-117X>), Romain François [aut] (<https://orcid.org/0000-0002-2444-4226>), Lionel Henry [aut], Kirill Müller [aut] (<https://orcid.org/0000-0002-1416-3412>), Davis Vaughan <davis@posit.co> [aut] (<https://orcid.org/0000-0003-4777-038X>) (2023). dplyr: A Grammar of Data Manipulation. R package version 1.1.3, <https://CRAN.R-project.org/package=dplyr>.
## 
## purrr, version 1.0.2, Hadley Wickham <hadley@rstudio.com> [aut, cre] (<https://orcid.org/0000-0003-4757-117X>), Lionel Henry <lionel@rstudio.com> [aut] (2023). purrr: Functional Programming Tools. R package version 1.0.2, <https://CRAN.R-project.org/package=purrr>.
## 
## readr, version 2.1.4, Hadley Wickham <hadley@posit.co> [aut], Jim Hester [aut], Jennifer Bryan <jenny@posit.co> [aut, cre] (<https://orcid.org/0000-0002-6983-2759>) (2023). readr: Read Rectangular Text Data. R package version 2.1.4, <https://CRAN.R-project.org/package=readr>.
## 
## tidyr, version 1.3.0, Hadley Wickham <hadley@posit.co> [aut, cre], Davis Vaughan <davis@posit.co> [aut], Maximilian Girlich [aut] (2023). tidyr: Tidy Messy Data. R package version 1.3.0, <https://CRAN.R-project.org/package=tidyr>.
## 
## tibble, version 3.2.1, Kirill Müller <kirill@cynkra.com> [aut, cre] (<https://orcid.org/0000-0002-1416-3412>), Hadley Wickham <hadley@rstudio.com> [aut] (2023). tibble: Simple Data Frames. R package version 3.2.1, <https://CRAN.R-project.org/package=tibble>.
## 
## ggplot2, version 3.5.2, Hadley Wickham (2016). ggplot2: Elegant Graphics for Data Analysis. R package version 3.5.2, <https://ggplot2.tidyverse.org>.
## 
## tidyverse, version 1.3.2, Hadley Wickham, Mara Averick, Jennifer Bryan, Winston Chang, Lucy D'Agostino McGowan, Romain François, Garrett Grolemund, Alex Hayes, Lionel Henry, Jim Hester, Max Kuhn, Thomas Lin Pedersen, Evan Miller, Stephan Milton Bache, Kirill Müller, Jeroen Ooms, David Robinson, Dana Paige Seidel, Vitalie Spinu, Kohske Takahashi, Davis Vaughan, Claus Wilke, Kara Woo, Hiroaki Yutani (2019). Welcome to the {tidyverse}. R package version 1.3.2, <https://CRAN.R-project.org/package=tidyverse>.
```

This document was generated using an R Markdown script adapted from Lena Matyjek, Muyu Lin and Andreea Tapuc, available under the MIT License. The original script is available at Open Science Framework.
